# Non-invasive stimulation reveals ventromedial prefrontal cortex function in reward prediction and reward processing

**DOI:** 10.3389/fnins.2023.1219029

**Published:** 2023-08-15

**Authors:** Maimu Alissa Rehbein, Thomas Kroker, Constantin Winker, Lena Ziehfreund, Anna Reschke, Jens Bölte, Miroslaw Wyczesany, Kati Roesmann, Ida Wessing, Markus Junghöfer

**Affiliations:** ^1^Institute for Biomagnetism and Biosignalanalysis, University Hospital Münster, Münster, Germany; ^2^Otto Creutzfeldt Center for Cognitive and Behavioral Neuroscience, University of Münster, Münster, Germany; ^3^Institute of Psychology, University of Münster, Münster, Germany; ^4^Institute of Psychology, Jagiellonian University, Kraków, Poland; ^5^Institute for Clinical Psychology, University of Siegen, Siegen, Germany; ^6^Department of Child and Adolescent Psychiatry, University Hospital Münster, Münster, Germany

**Keywords:** reward prediction, reward processing, prediction error, ventromedial prefrontal cortex, transcranial direct current stimulation, magnetoencephalography

## Abstract

**Introduction:**

Studies suggest an involvement of the ventromedial prefrontal cortex (vmPFC) in reward prediction and processing, with reward-based learning relying on neural activity in response to unpredicted rewards or non-rewards (reward prediction error, RPE). Here, we investigated the causal role of the vmPFC in reward prediction, processing, and RPE signaling by transiently modulating vmPFC excitability using transcranial Direct Current Stimulation (tDCS).

**Methods:**

Participants received excitatory or inhibitory tDCS of the vmPFC before completing a gambling task, in which cues signaled varying reward probabilities and symbols provided feedback on monetary gain or loss. We collected self-reported and evaluative data on reward prediction and processing. In addition, cue-locked and feedback-locked neural activity via magnetoencephalography (MEG) and pupil diameter using eye-tracking were recorded.

**Results:**

Regarding reward prediction (cue-locked analysis), vmPFC excitation (versus inhibition) resulted in increased prefrontal activation preceding loss predictions, increased pupil dilations, and tentatively more optimistic reward predictions. Regarding reward processing (feedback-locked analysis), vmPFC excitation (versus inhibition) resulted in increased pleasantness, increased vmPFC activation, especially for unpredicted gains (i.e., gain RPEs), decreased perseveration in choice behavior after negative feedback, and increased pupil dilations.

**Discussion:**

Our results support the pivotal role of the vmPFC in reward prediction and processing. Furthermore, they suggest that transient vmPFC excitation via tDCS induces a positive bias into the reward system that leads to enhanced anticipation and appraisal of positive outcomes and improves reward-based learning, as indicated by greater behavioral flexibility after losses and unpredicted outcomes, which can be seen as an improved reaction to the received feedback.

## 1. Introduction

Striving for reward appears to promote evolutionary survival. To acquire a reward, one needs to be able to predict the reward value of a stimulus, process this reward value, and learn from experience (Schultz, 2015). Research in non-human primates has shown that reward-based learning relies heavily on reward prediction error (RPE). An unexpected reward results in increased activity (+RPE) while unexpected non-rewards—i.e., when a reward was predicted, but did not occur—results in a decreased activity (-RPE; e.g., Mirenowicz and Schultz, 1996; Schultz, 1998, 2016).

In humans, time-sensitive electroencephalography (EEG) or magnetoencephalography (MEG) has revealed a characteristic neural response to reward that encompasses the so-called reward positivity (RewP; Reward/Gain vs. Punishment/Loss; Proudfit, 2015) and the subsequent P300. The RewP is also known as feedback negativity, feedback-related negativity, or feedback-error-related negativity (FN, FRN, FERN; Punishment/Loss vs. Reward/Gain; Miltner et al., 1997; Gehring and Willoughby, 2002). The RewP emerges around 250 ms after feedback presentation as a frontocentral positivity for trials in which feedback indicates a relative gain (e.g., monetary reward) as compared to a relative loss (e.g., monetary punishment; e.g., Foti et al., 2011). The neuroelectric RewP is sensitive to RPEs, with a higher magnitude for unpredicted as compared to predicted outcomes (but see Hajcak et al., 2005; Holroyd et al., 2009). Moreover, it is not only observed in response to feedback but also in response to cues that predict the occurrence or non-occurrence of reward (Holroyd et al., 2011). Source localization of the RewP frequently points to the anterior cingulate cortex (ACC) and sometimes to the ventromedial prefrontal cortex (vmPFC) or to the striatum (Gehring and Willoughby, 2002; Carlson et al., 2011; Potts et al., 2011; see Walsh and Anderson, 2012, for a review). The RewP can also indicate aberrant reward processing in psychopathology. Parvaz et al. (2015), for example, observed impairments of RewP modulation and RPEs in individuals with substance-use disorder during a gambling task with varying reward probabilities. Patients showed a differing FN/RewP after unpredicted compared to predicted outcomes. While cocaine-positive addicts were lacking a modulation of the FN/RewP by prediction only after losses, abstinent cocaine addicts did not show an effect of prediction on the FN/RewP at all (for gains and losses). One might infer that addicts' reinforcement learning capability is diminished, especially after losses or (prediction) errors ultimately resulting in difficulties in adapting behavior accordingly (Parvaz et al., 2015). The neuromagnetic signature to reward—though less often investigated—resembles the neuroelectric one with regard to temporal development and sensitivity to reward prediction error (RPE; Doñamayor et al., 2012; Talmi et al., 2012).

Central structures of the brain's reward system are the striatum, midbrain dopamine neurons, and prefrontal cortex, including ACC and vmPFC (see Haber and Knutson, 2010, for a review). The vmPFC cannot be regarded as an anatomical structure with clearly defined borders, but rather as a brain region within the lower half of the mPFC and the medial part of the orbitofrontal cortex (Kringelbach, 2005; Myers-Schulz and Koenigs, 2012; Schneider and Koenigs, 2017; Hiser and Koenigs, 2018). Bidirectional pathways connect the vmPFC to numerous brain regions, such as the amygdala, other structures within the frontal cortex, or the cortico-striatal-thalamic loop (see Euston et al., 2012, for an overview).

Based on previous findings (e.g., Knutson et al., 2001, 2003; Cao et al., 2019), Schneider and Koenigs (2017) suggested that the vmPFC monitors and updates the reward value of a stimulus. Furthermore, vmPFC activation reflects individual differences in the expectation and the processing of rewards (Knutson et al., 2005). For instance, the optimism bias (Sharot, 2011), the overestimation of reward probability or positive outcomes (Sharot, 2011), has been associated with the activation of several brain regions, including the vmPFC (Sharot et al., 2007; Blair et al., 2013). In addition, vmPFC regions appear specifically responsive to the evaluation of positive relative to negative scenes (Sabatinelli et al., 2007), an effect not observed in dysphoric individuals (Sabatinelli et al., 2015).

Although previous studies using different methodologies have shown the importance of the vmPFC within the reward network, its exact role in predicting and processing rewards as well as RPE signaling remains unclear. Investigations that transiently modulate vmPFC excitability in a gambling setting could contribute to understanding vmPFC function within the reward system, as this direct activity modulation allows us to draw causal conclusions about the underlying network. Transcranial direct current stimulation (tDCS) provides a suitable approach for the transient up- or downregulation of vmPFC activity. Anodal or excitatory tDCS depolarizes the membrane potential of cells and thereby increases the excitability of neurons. Cathodal or inhibitory tDCS hyperpolarizes the membrane potential and thereby decreases neural excitability (Sparing and Mottaghy, 2008).

In previous fMRI and MEG studies of our group, we have demonstrated that excitatory relative to inhibitory tDCS of the vmPFC biases the processing of emotional scenes and emotional facial expressions on the behavioral and the neural level toward a relative preference for positive as compared to negative emotional material, consistent with a positivity bias (Junghofer et al., 2017; Winker et al., 2018, 2019, 2020; Kroker et al., 2022). Importantly, other groups also report modulation of the reward positivity (i.e., positive stimulus processing) by non-invasive stimulation of the medial prefrontal cortex (Ryan et al., 2022).

In the present study, we aimed at investigating the causal role of the vmPFC in the prediction and processing of reward. To this end, participants performed a gambling task with varying reward probabilities on 2 separate days. Directly before the task, participants received either excitatory or inhibitory non-invasive tDCS of the vmPFC. During this gambling task, participants were asked to gamble on various trials, in which a cue signaled the respective reward probability (i.e., 33, 50, or 67%) and a colored symbol provided feedback on the reward (i.e., monetary gain) or punishment (i.e., monetary loss). As dependent variables, we collected self-report and evaluative data of reward prediction and reward processing in addition to recording cue-locked (i.e., before participants received the feedback) and feedback-locked (i.e., after participants received the feedback) neural activity and pupil diameter via MEG and eye-tracking, respectively. Pupil recordings were implemented because pupil data are an important correlate in reward prediction as they are associated with decision-making (Kozunova et al., 2021). Additionally, pupil dilation increases after rewards especially when rewards were occurring reliably (Lavín et al., 2014).

With regard to reward prediction (cue), we expected to observe a general overestimation of reward probability in the self-report as well as enhanced neural responses especially in salience-sensitive brain areas, developing in early (<300 ms) and later components (>300 ms), and increased pupil diameters in trials with greater actual or expected reward probability, irrespective of tDCS. With regard to reward processing (feedback), we expected to observe more positive evaluations as well as enhanced neural processing especially in salience-sensitive brain areas, developing in early (<300 ms) and later components (>300 ms), and pupil diameters in trials with rewarding outcome, irrespective of tDCS. With regard to the effects of vmPFC-tDCS on reward prediction, we expected to observe a greater overestimation of reward probability in the self-report as well as greater differential responses in neural measures and pupil diameter in trials with greater actual or expected reward probability after vmPFC excitation as compared to inhibition (consistent with an enhanced optimism bias after vmPFC excitation). With regard to the effects of vmPFC-tDCS on reward processing, we expected to observe more positive evaluations and greater differential responses in neural measures and pupil diameter in trials with rewarding outcomes after vmPFC excitation as compared to inhibition (consistent with an enhanced positivity bias after vmPFC excitation). Importantly, we expected the influence of tDCS-induced changes in vmPFC activity on reward prediction and reward processing to not function independently. Instead, we predicted that vmPFC excitation as compared to inhibition should enhance responses, especially in those trials in which the feedback deviates from the prediction, thereby modulating RPEs.

## 2. Materials and methods

### 2.1. Participants

A total of 41 (21 female) right-handed volunteers, aged 19–33 years (*M* = 23.85, *SD* = 3.25), participated in this study, which was conducted at the Institute for Biomagnetism and Biosignal Analysis in Münster, Germany (see Table 1). Participants were recruited via social media and the participant pool of the institute. These 41 participants had no current or lifetime psychological disorders, did not undergo psychotherapy or psychopharmacological treatment (currently or in the past), had no neurological or severe somatic disease, no removable metal on head or neck, no red-green color vision deficiency (see Ishihara's test below), a sum score of 11 and lower in the Beck-Depression-Inventory-II (BDI-II; Beck et al., 1996), did not participate in a similar study at the institute, or were pregnant.

**Table 1:** Characteristics of participants.

	**M**	**SD**	***χ^2^***	**df**	***p***
**Demographic characteristics**					
*N*	41				
Female (*N*, %)	21 (51.2)	-	*0.02*	1	0.876
Stimulation order (Exc-Inh, %)	21 (51.2)	-	*0.02*	1	0.876
Age (years)	23.90	3.16			
**Psychometric characteristics**					
BDI-II	1.54	2.26			
RR	25.30	3.15			
UI-18	36.85	11.48			
SDS	14.32	4.15			

Stimulation order—Excitatory first, Inhibitory Second: N = 21; Inhibitory first, Excitatory second: N = 20.

BDI-II, Beck's Depression Inventory (Beck et al., 1996); RR, Scale for Measuring Reward Responsiveness (Van den Berg et al., 2010); UI-18, Intolerance of Uncertainty scale (Gerlach et al., 2008); SDS-CM, Social Desirability Scale (Crowne and Marlowe, 1960).

The italic values indicate chi-squared values, comparable to t- or F-values.

To ensure authentic reactions to the gambling task, participants were told a cover story. They were instructed that they could win an amount ranging between 0.00 and 36.00 € depending on their performance in the task, in addition to receiving a basic allowance of 30.00 €. Having completed the study, all participants received the full amount of 66.00 € and were informed of the actual reward contingency.

The experimental procedure complied with the directives of the Helsinki Declaration and was approved by the ethics committee of the medical faculty of the University of Münster (2019-251-f-S).

### 2.2. Gambling task

**Figure 1:**
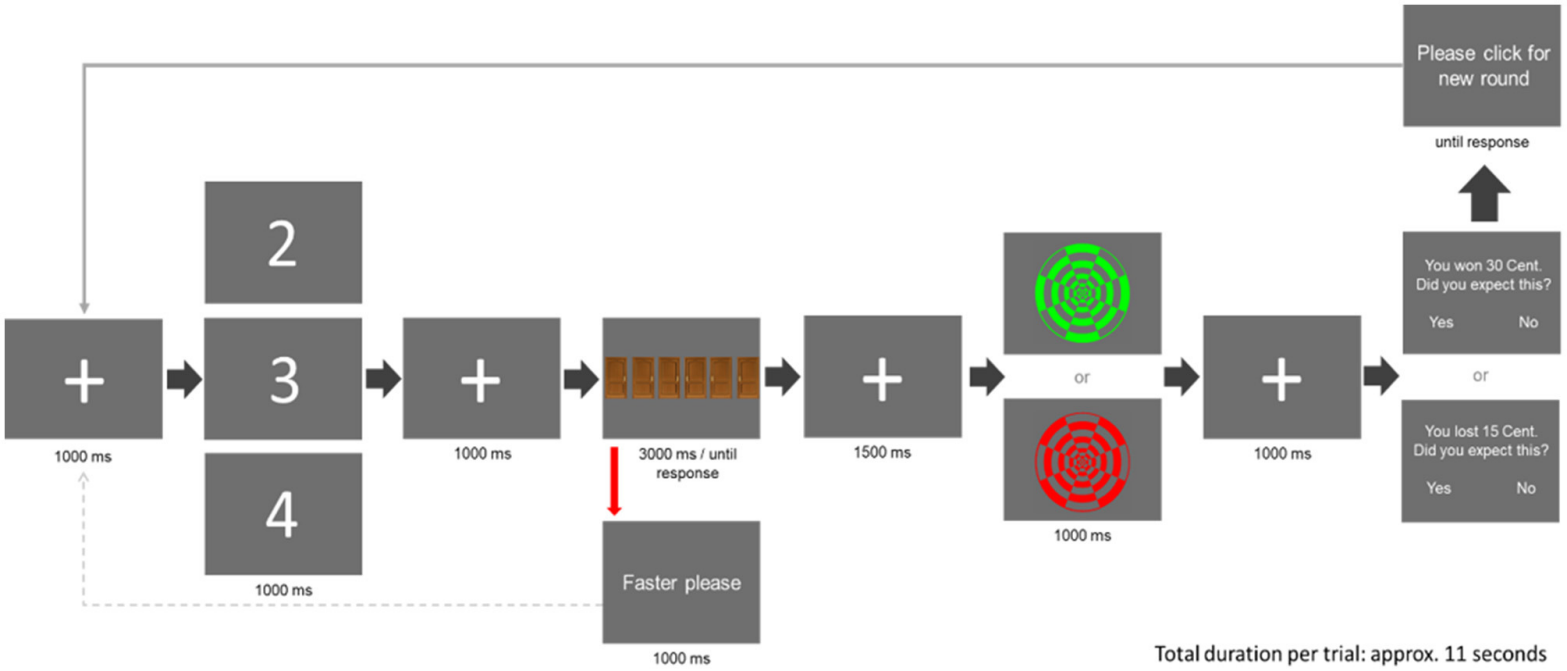
The course of one trial in the gambling task (adapted from Parvaz et al., 2015). Each trial started with the presentation of a fixation cross, followed by a cue (“2,” “3,” or “4”). Participants were told that the cue indicated the number of doors, behind which a monetary “gain” was hidden, i.e., the reward probability. Following another fixation cross, six doors came on display out of which participants were asked to choose one. After the selection of one door, a feedback stimulus (“gain” or “loss”) was presented. As feedback, either a green concentric circle, indicating a monetary gain of 30 cents, or a red concentric circle, indicating a monetary loss of 15 cents, was shown. After that, the outcome (gain and loss) was repeated and participants were asked to indicate whether they had or had not predicted this outcome.

The gambling task (see Figure 1) was adapted from Parvaz et al. (2015). At the beginning of each trial, a fixation cross was shown (1,000 ms duration), followed by a cue, displaying the numbers “2,” “3,” or “4” (1,000 ms duration). Participants were told that the cue indicated the number of doors (two, three, or four out of six, respectively), behind which a monetary gain was hidden, i.e., the reward probability (33, 50, and 67%). Each cue occurred 80 times in a pseudorandomized order ensuring that no cue appeared more than twice in a row. The cue was followed by another fixation cross (1,000 ms duration), preceding the display of the six doors. Participants had to choose one of the six doors via a response box using the index finger of their right hand. If no door was chosen for 3,000 ms, they were instructed to respond faster, before the doors were displayed again. After a sufficiently fast response, another fixation cross was shown (1,500 ms duration), followed by the presentation of the feedback stimulus (1,000 ms duration). As feedback stimuli, either a green or a red circular checkerboard pattern of identical brightness was displayed. The dark-bright contrast pattern was reversed between the two stimuli so that discrimination was possible not only based on color but also based on contrast (balanced across participants). Participants were told that feedback depended on their choice, with green indicating a monetary gain following “correct” choices and red indicating a monetary loss following “wrong” choices. However, irrespective of the subjects' choice, all participants were shown gain outcomes in 28 out of 80 trials following cue “2” (reward probability = 35%), in 40 out of 80 trials following cue “3” (reward probability = 50%), and in 52 out of 80 trials following cue “4” (reward probability = 65%),^1^ with the array of gains and losses randomized across participants. Following the presentation of the outcome, another fixation cross was presented (1,000 ms duration). Subsequently, participants were reminded of the previous outcome (gain and loss) and were asked to rate whether they had predicted it (“You have won 30 cents/You have lost 15 cents. Did you predict that? Yes or No”). As in Proudfit (2015), the amount of money to be won (30 cents) was double the amount to be lost (15 cents), since losses correspond to approximately twice the emotional value of gains (Kahneman and Tversky, 1979; Tversky and Kahneman, 1992). The gambling task was programmed using the Psychophysics Toolbox Version 3 (http://psychtoolbox.org).

### 2.3. tDCS

**Figure 2:**
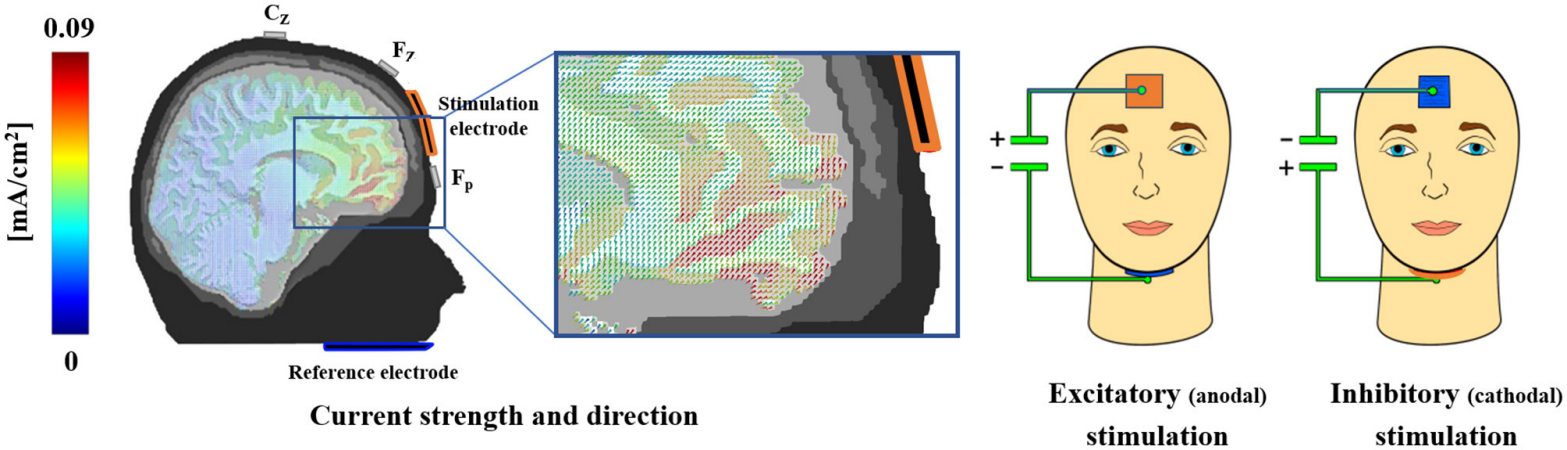
Electrode montage and finite element-based modeling of tDCS currents. The volume conductor model implies maximal anterior vmPFC-stimulation with the adapted electrode montage on the forehead and chin, while adjacent brain regions receive minimal stimulation. For excitatory (anodal) or inhibitory (cathodal) stimulation of the vmPFC, the electrode positioned on the forehead was used as the anode or cathode, respectively. For visualization purposes, sponges are displayed here in different colors, i.e., red representing the anode and blue representing the cathode. However, sponges and cables of blue color were used for both stimulation sessions to prevent participants from inferring different stimulation conditions. This figure was published first by Junghofer et al. (2017) *Cerebral Cortex*. Authorization to republish is available.

tDC stimulation of the vmPFC was performed consistent with the protocol used in our previous studies (Junghofer et al., 2017; Winker et al., 2018, 2019, 2020; Roesmann et al., 2021; Kroker et al., 2022; see Figure 2). One stimulation electrode patch (3 × 3 cm) was positioned on the forehead and the other bigger patch (5 × 5 cm), serving as an extracephalic reference, was positioned under the chin. Both electrodes were inserted into sponges, which were soaked in a sodium chloride solution to ensure electric conductivity. For excitatory (anodal) or inhibitory (cathodal) stimulation of the vmPFC, the electrode on the forehead was used as the anode or cathode, respectively. Finite element-based forward modeling of tDCS currents revealed that this electrode composition leads to maximal stimulation of the anterior vmPFC and minimal stimulation of other brain regions (Wagner et al., 2014). A DC Stimulator Plus was used (NeuroConn GmbH, Ilmenau, Germany) to apply a current of 1.5 mA for 10 min, with 10 s fade-in and fade-out phases.

### 2.4. Experimental procedure

**Figure 3:**
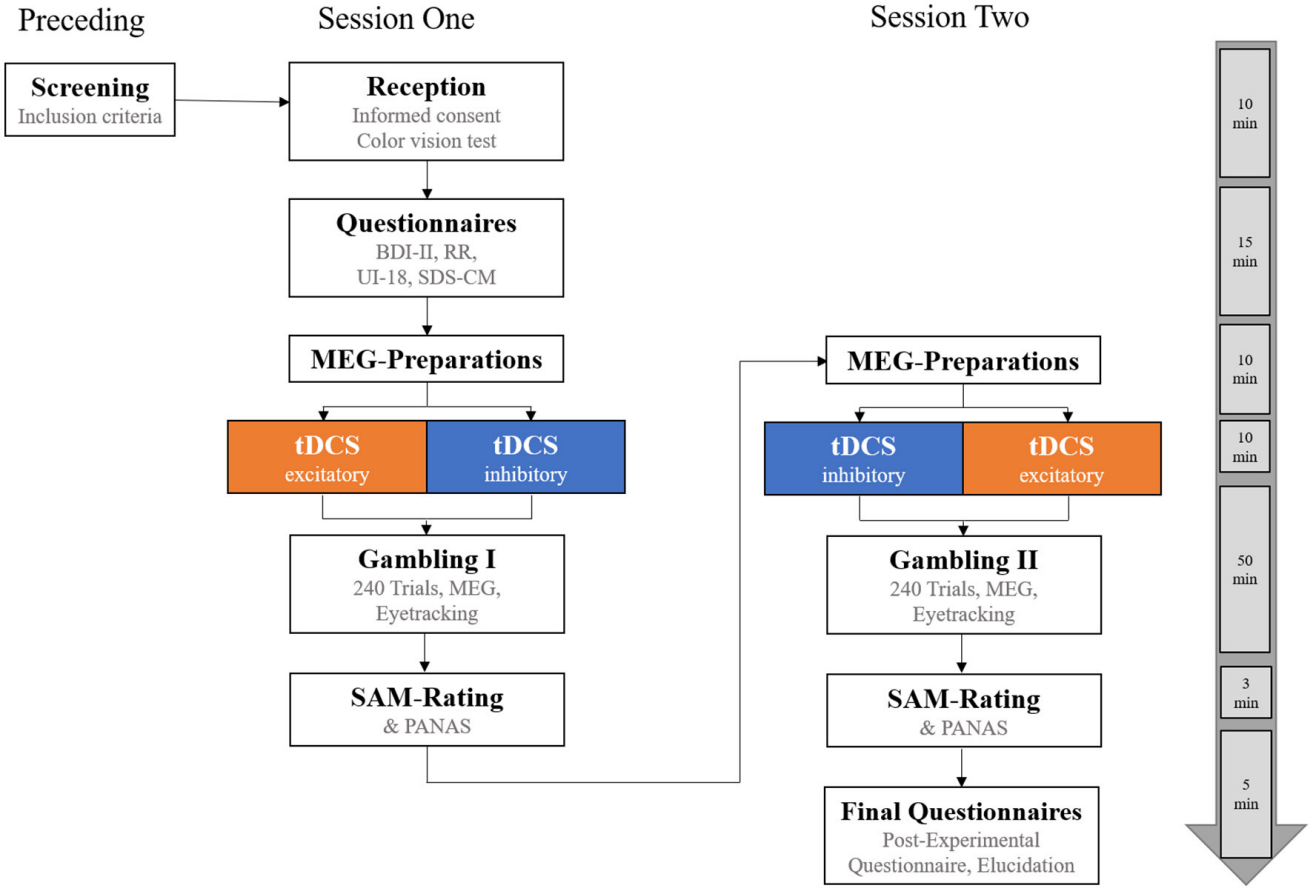
Overview of the experimental procedure. Participants completed two experimental sessions on 2 different days with a minimum of 48 h between the sessions. They were pseudorandomly allocated to one of two stimulation orders, receiving either excitatory transcranial Direct Current Stimulation (tDCS) of the vmPFC before gambling in session one and inhibitory tDCS in session two, or vice versa. On the far right, an approximate timeline for the experimental procedure is shown. BDI-II, Beck Depression Inventory-II; RR, Scale for Measuring Reward Responsiveness; UI-18, Intolerance of Uncertainty scale; SDS-CM, Social Desirability Scale by Crowne and Marlowe; SAM-Rating, Subjective Ratings of Hedonic Valence and Emotional Arousal; PANAS, Positive and Negative Affect Schedule.

All participants took part in two experimental sessions conducted on two different days (see Figure 3). At the beginning of the first session, participants gave written informed consent. They were checked for accurate color vision (for undisturbed differentiation of the red/green feedback stimuli) by Ishihara's test for red-green color deficiency (Clark, 1924) and completed the following questionnaires: the Beck Depression Inventory (BDI-II; Beck et al., 1996), the Reward Responsiveness scale (RR; Van den Berg et al., 2010), the Intolerance of Uncertainty scale (UI-18; Gerlach et al., 2008), and the Social Desirability Scale (SDS-CM; Crowne and Marlowe, 1960). Subsequently, participants received either excitatory or inhibitory vmPFC-tDCS for 10 min. Immediately following stimulation (<5 min after the end of stimulation), they performed the gambling task in the MEG scanner, which consisted of 240 trials split into four blocks of 60 trials each (with short breaks in between and a total duration of ~45 min). Afterward, participants rated the gain and loss stimuli with regard to the subjective hedonic valence and emotional arousal on a 9-point digitized self-assessment manikin (SAM) rating scale (Bradley and Lang, 1994). Finally, to track possible changes in mood as a result of tDCS, participants were also asked to complete the Positive and Negative Affect Schedule (PANAS; Watson et al., 1988). After the entire procedure, participants were educated about the cover story.

Each participant received an excitatory and an inhibitory stimulation for two sessions (minimum interval of 48 h between the sessions), but stimulation order was counterbalanced across subjects (excitatory tDCS in session one and inhibitory tDCS in session two, *N* = 21, or vice versa, *N* = 20).

Participants allocated to the two stimulation orders did not differ with regard to gender, age, BDI-II, RR, UI-18, and SDS (*p*-values > 0.350). Following tDCS in the second experimental session, participants again performed the gambling task and completed the SAM-ratings and PANAS as in the first experimental session. Finally, participants were elucidated about the cover story. Altogether, the experiment lasted for about 200 min (session 1: 110 min; and session 2: 90 min).

### 2.5. Recording and preprocessing of MEG

Using the 275 whole-head sensor system with first-order axial gradiometers (Omega 275; CTF, VSM MedTech Ltd., Coquitlam, Canada), we measured visually evoked magnetic fields (VEMFs) in a frequency range between 0 and 150 Hz with a sampling rate of 600 Hz. Head position in the scanner and head movement during measurement were registered using landmark coils, which were positioned on the nasion and in both earlobes. Offline, a 48-Hz low-pass and 0.1 Hz high-pass filter were employed and data were sampled down to 300 Hz. Epochs from 200 ms before to 600 ms after stimulus onset were extracted and baseline-adjusted, using a −150 to 0 ms (i.e., stimulus onset) interval. In the case of assessing reward prediction, stimulus onset corresponded to the onset of the cue (“2,” “3,” and “4;” see Figure 1). In the case of assessing reward processing, stimulus onset corresponded to the onset of the feedback (gain, loss; concentric circles in Figure 1). Single trials were edited using an established method for the statistical control of artifacts in high-density electro- and magneto-encephalography data (Junghöfer et al., 2000). In line with this method, artifacts in individual channels as well as global artifacts were identified and signals of artifact-contaminated channels were replaced by spherical spline interpolation based on the signal of all remaining channels. A minimum threshold of 0.01 for the Goodness of Interpolation was employed (testing the interpolation of 275 test topographies based on the residual sensor configuration in each trial) and trials exceeding this value were rejected. Because more than 30% of trials in any MEG run were rejected, five participants were excluded from further analysis. Neural responses were averaged across trials and the four MEG runs, separately for each participant, stimulation session (excitatory and inhibitory), cue (“2,” “3,” and “4”) in the case of cue-locked responses, or outcome (gain and loss) in the case of feedback-locked responses. Considering the subsequent self-reported reward prediction (i.e., participants' prediction of the respective outcome at the end of each trial), additional averages were calculated in the case of feedback-locked responses, corresponding to the combinations of predicted outcome with the received outcome (gain predicted/gain received, gain predicted/loss received, loss predicted/gain received, and loss predicted/loss received). In the case of cue-locked responses, additional averages were also calculated for the predicted outcome (gain predicted and loss predicted) across all cues. However, the number of trials did not allow for averaging with respect to the combinations of predicted outcome and cue.

Underlying sources of measured magnetic fields were inversely modeled by use of the L2-Minimum-Norm estimation (L2-MNE; Hämäläinen and Ilmoniemi, 1994). *A priori* assumptions about the location and distribution of active dipoles are not needed for this model. A spherical model with 350 evenly distributed dipole pairs (azimuthal and polar direction) and a source shell radius approximately corresponding to the gray matter depth (i.e., 87% of the individually fitted head) was employed as a source model. Topographies of the L2-MNE were established with a Tikhonov regularization parameter of *k* = 0.1. The source-direction-independent neural activities (vector length of the estimated source activities at each position) were calculated for each individual participant, condition, and time point. In addition to the five participants excluded during artifact inspection, further three participants were excluded in a subsequent outlier analysis conducted in source space. In these participants, either the mean of the standard deviation between experimental conditions across time or the maximum of normed (by mean) standard deviation between experimental conditions differed from the sample median by more than four standard deviations. This resulted in the final sample of 33 participants for analysis of MEG data, which was counterbalanced regarding sex and stimulation order as well (*p* > 0.800).

### 2.6. Recording and preprocessing of pupil data

Pupil dilations in response to cues and outcomes were measured with a sampling rate of 600 Hz using an eye tracker (EyeLink 1000 Plus; SR Research Ltd., Canada). Epochs ranging from 200 ms before to 1,800 ms after cue onset and after feedback onset were extracted. The interval from −150 to 0 ms (i.e., cue or feedback onset) served for baseline adjustment. Pupil data were sampled down to 300 Hz, filtered, baseline corrected, and averaged across trials, separately for each participant and condition. Preprocessing of the pupil data was performed as in the MEG. Due to an outlier analysis, four participants of the MEG sample had to be excluded as their mean number of valid trials across conditions was four standard deviations below the median sample trial number. This resulted in a final sample of 29 participants for analysis of pupil data. To keep the physiological effects as comparable as possible, subjects whose MEG data were not usable were also excluded from this analysis.

### 2.7. Analysis of reward prediction and its modulation via vmPFC-tDCS

#### 2.7.1. Behavioral correlates

During each trial of the gambling task, we collected self-reported reward predictions, asking participants to indicate whether they had or had not predicted a gain or a loss outcome to occur in the respective trial. The self-report was recoded into a binary response variable, i.e., predicted outcome (gain predicted and loss predicted), because of which chi-square tests and logistic regressions were computed. Using the chi-squared test, we examined whether participants overestimated the probability of winning regardless of the stimulation condition. The influence of the predictor's REWARD PROBABILITY (33, 50, and 67%) and STIMULATION (excitatory and inhibitory) on the relative frequency of reward predictions in the self-report was evaluated in logistic regression. Here, all subjects could be included.

#### 2.7.2. Neural correlates

We included cue-locked responses for the following analysis of neural correlates. First, we calculated an ANOVA with the factors PREDICTED OUTCOME (gain predicted and loss predicted) and STIMULATION (excitatory and inhibitory), analogous to the behavioral analysis. Second, we calculated an ANOVA with the factors REWARD PROBABILITY (33, 50, and 67%) and STIMULATION (excitatory and inhibitory). The factors PREDICTED OUTCOME and REWARD PROBABILITY were not included in the same analysis to maintain a sufficient signal-to-noise-ratio.^2^

We applied intervals of interest from 0 to 300 ms to investigate early bottom-up processes and 300 to 600 ms for later cognitive processes, as we did in previous studies (Winker et al., 2018; Roesmann et al., 2021; Kroker et al., 2022). If any effect overlapped the interval limit of 300 ms, the interval was extended stepwise by 50 ms. Taking the problem of multiple comparisons into account, we applied a non-parametric correction method (Maris and Oostenveld, 2007). More specifically, the statistical value of each time point and dipole was tested for significance with an α-level of *p* = 0.05 (sensor-level criterion). A cluster mass was calculated by the spatio-temporal integral of the statistical value, the spatial extent, and the temporal extent. Then, the cluster mass was tested against 1,000 permuted random drawings, which were drawn from the original data set as well. The distribution of the permutations was employed to determine the cluster criterion. If a cluster mass exceeded the critical cluster mass of *p* = 0.05 (i.e., >95% of the permuted clusters; cluster-level criterion), the effect was classified as significant. To avoid false temporal precision, cluster onset and offset were rounded to the nearest 10 ms. This procedure was implemented for all examined effects. For visualization purposes, L2-MNE topographies were projected onto standard 3D brain models.

#### 2.7.3. Autonomous nervous system (ANS) correlates

Pupil diameter was employed as a measure of ANS activity. Again, the analysis focused on cue-locked responses. Statistical analysis of pupil data was similar to the analysis of MEG data, except that only one sensor was examined. Thus, the statistical analysis resulted in a temporal cluster (but not a spatio-temporal cluster), as the cluster mass was calculated as the temporal integral of the statistical values. We employed the same criteria as in the MEG (sensor-level: 0.05, cluster-level: 0.05) to test the respective effects. To be consistent with the MEG analysis, we calculated an ANOVA including the factors PREDICTED OUTCOME (gain predicted and loss predicted) and STIMULATION (excitatory and inhibitory) and an ANOVA with the factors REWARD PROBABILITY (33, 50, and 67%) and STIMULATION (excitatory and inhibitory).

### 2.8. Analysis of reward processing and its modulation via vmPFC-tDCS

#### 2.8.1. Behavioral correlates

One participant was excluded due to misunderstanding the questionnaires and two further subjects were excluded due to missing values resulting in a final sample of 38 participants. The subjective ratings of hedonic valence and emotional arousal, which were collected once for each feedback stimulus at the end of each session (not trial-wise), were analyzed via ANOVAs with the factors FEEDBACK (gain feedback and loss feedback) and STIMULATION (excitatory and inhibitory).

#### 2.8.2. Neural correlates

Here, we were interested in feedback-locked neural responses. We calculated an ANOVA with the factors RECEIVED OUTCOME (gain received and loss received), PREDICTED OUTCOME (gain predicted and loss predicted), and STIMULATION (excitatory and inhibitory). Since strong main effects of the feedback (loss > gain; see Figure 9A) masked weaker effects (gain > loss; see Figure 9B) by explaining most of the variance in the ANOVA (probably due to perceptual differences), we calculated a paired *t*-test to analyze influences of RECEIVED OUTCOME on neural responses. This allows us to differentiate positive (gain > loss) from negative effects (loss > gain).

Intervals of interest and correction for multiple comparisons were applied consistent with the analysis of reward prediction.

#### 2.8.3. ANS correlates

To analyze feedback-locked pupil responses, we again calculated an ANOVA with the factors RECEIVED OUTCOME (gain received and loss received), PREDICTED OUTCOME (gain predicted and loss predicted), and STIMULATION (excitatory and inhibitory).

### 2.9. Analysis of mood effects

To capture potential changes in the mood of participants induced by tDCS, we analyzed the PANAS scores by calculating an ANOVA with the factors AFFECT (positive and negative) and STIMULATION (excitatory and inhibitory).

Pre-processing and analysis of MEG and pupil data were carried out using the MATLAB (version 2019b)-based Electromagnetic Encephalography Software EMEGS (version 3.2; Peyk et al., 2011). Behavioral and questionnaire data as well as subjective ratings of hedonic valence and emotional arousal were analyzed employing the statistics program R (2015), applying a significance level of α = 0.05. ANOVAs for repeated measures were used, unless otherwise specified. All behavioral and neural effects were qualitatively equivalent if the smallest shared sample of 29 participants was used.

## 3. Results

### 3.1. Reward prediction and its modulation via vmPFC-tDCS

#### 3.1.1. Behavioral correlates

**Figure 4:**
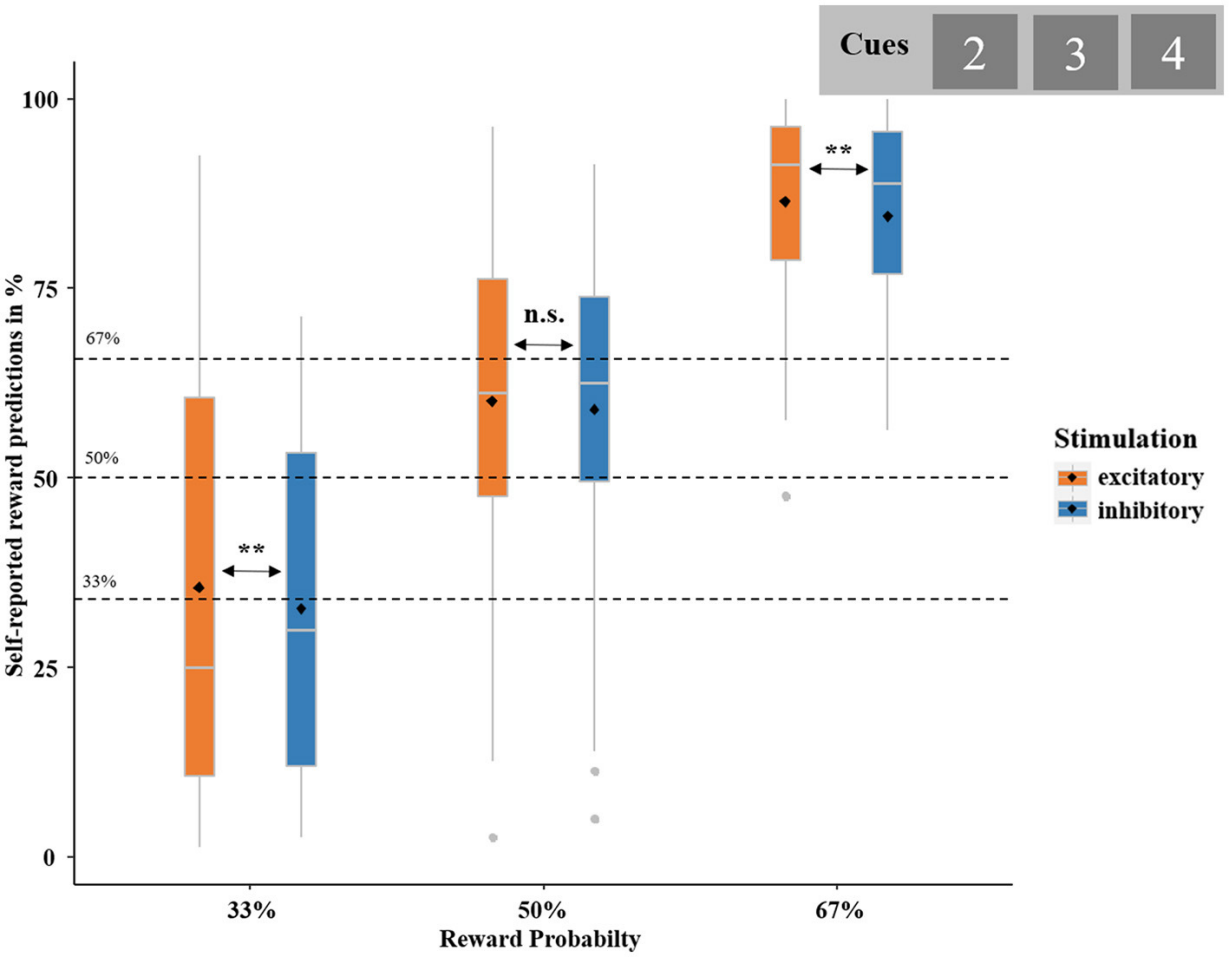
Self-reported reward predictions in percent. Boxplots represent the probability of expecting to receive a reward, calculated based on participants' self-report at the end of each trial (“Did you expect this outcome?”). Self-reported reward predictions are broken down by actual REWARD PROBABILITY (i.e., 33, 50, and 67%; dashed black lines)—as indicated by cue value (“2,” “3,” and “4”)—and STIMULATION (excitatory and inhibitory). Boxplots indicate means (black dot), medians (gray line), and lower and upper quartiles. Asterisks indicate significance levels: + <0.1, * <0.05, ** <0.01, *** <0.001.

The chi-squared test comparing the observed proportion of predicted gains and the reward probability (average of $\frac{1}{3}$, $\frac{1}{2}$ and $\frac{2}{3}$ = $\frac{1}{2}$) conweward probability $\chi {{\;}^{\text{2}}}_{\left(1\right)}$ = 688.47, *p* < 0.001, *W* = 2.43, which increased with increasing reward probability, $\chi {{\;}^{\text{2}}}_{\left(2\right)}$ = 11.45, *p* = 0.003, $\eta_{\left(H\right)}^{2}$ = 0.08 (*post-hoc* tests: 33 vs. 50%: $\chi {{\;}^{\text{2}}}_{\left(1\right)}$ = 2.94, *p* = 0.087, $\eta_{\left(H\right)}^{2}$ = 0.03; 50 vs. 67%: $\chi {{\;}^{\text{2}}}_{\left(1\right)}$ = 3.54, *p* = 0.059, $\eta_{\left(H\right)}^{2}$ = 0.04; 33 vs. 67%: $\chi {{\;}^{\text{2}}}_{\left(1\right)}$ = 10.65, *p* = 0.001, $\eta_{\left(H\right)}^{2}$ = 0.13; see Figure 4). The logistic regression yielded the significance of the model including the predictors REWARD PROBABILITY and STIMULATION on self-reported reward predictions, $\chi {{\;}^{\text{2}}}_{\left(3\right)}$ = 52.83, *p* < 0.001. This revealed a significant influence of REWARD PROBABILITY (*z* = 5.47, *p* < 0.001, *OR* = 2.66), but not STIMULATION (*z* = −0.65, *p* = 0.511) or the interaction of REWARD PROBABILITY by STIMULATION (*z* = −0.05, *p* = 0.964). However, based on our specific hypothesis on the influence of stimulation on predictions, we computed a second logistic regression with STIMULATION (excitatory, inhibitory) as the unique predictor. This analysis showed that reward prediction was greater after excitatory tDCS than after inhibitory tDCS [$\chi {{\;}^{\text{2}}}_{\left(1\right)}$ = 5.67, *p* = 0.017, *OR* = 0.87]. As hypothesized, participants generally overestimated the probability of receiving a reward. This overestimation occurred for the 50% condition and was even stronger for the 67% condition. Importantly, excitatory compared to inhibitory vmPFC stimulation resulted in an increased overestimation in the conditions with the lowest (33%) and highest (67%) probability.

#### 3.1.2. Neural correlates (cue-locked)

##### 3.1.2.1. PREDICTED OUTCOME-by-STIMULATION ANOVA

**Figure 5:**
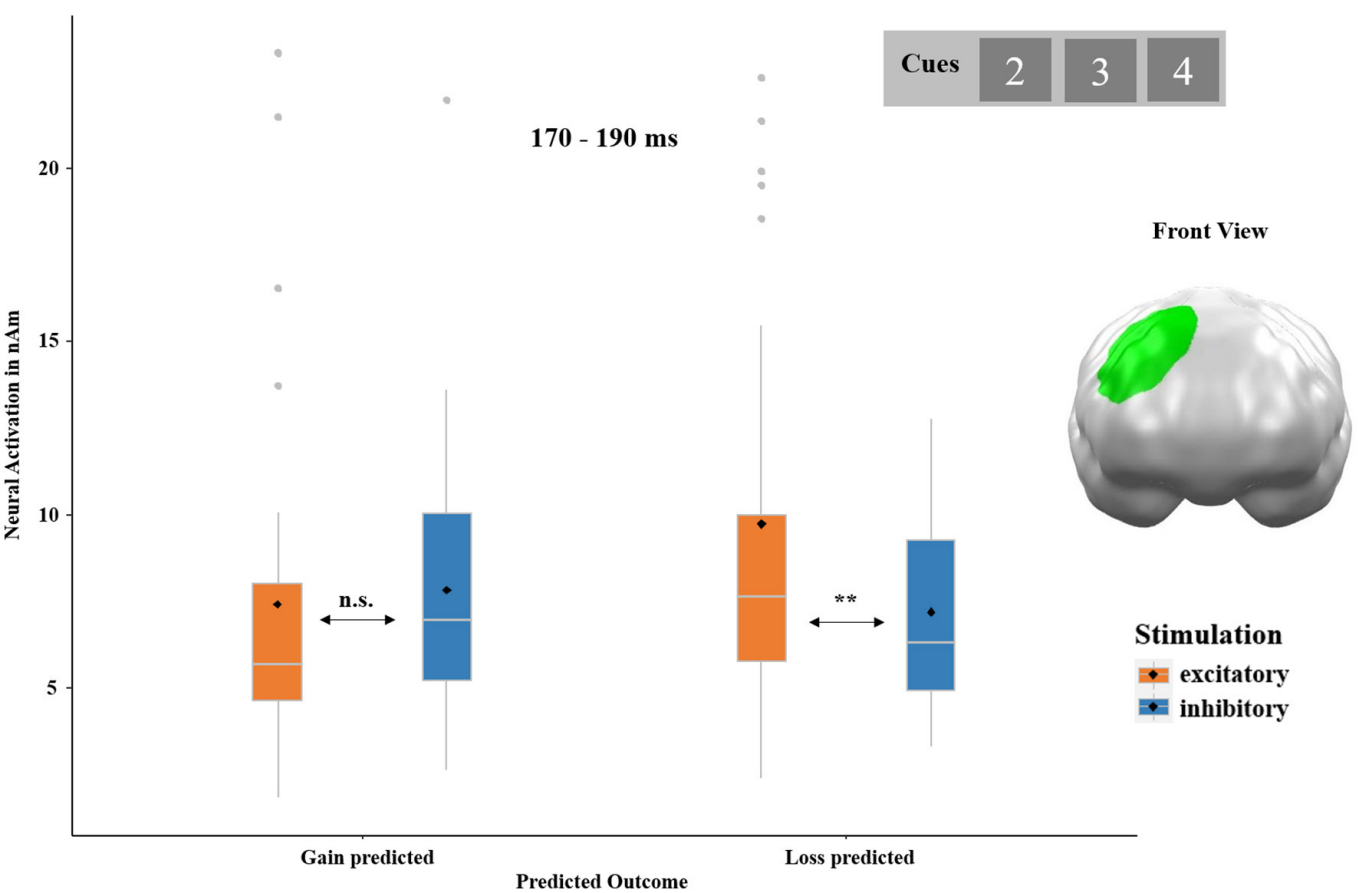
Significant spatio-temporal cluster featuring an interaction effect of PREDICTED OUTCOME (gain predicted and loss predicted) and STIMULATION (excitatory and inhibitory) on cue-locked neural responses in a right dorsolateral prefrontal cortex (dlPFC) region between 170 and 190 ms. Cues preceding the prediction of a loss evoked stronger neural responses after excitatory as compared to after inhibitory stimulation. Topographies of effects observed in L2-MNE were projected on standard 3D brain models for visualization. Boxplots indicate means (black dot), medians (gray line), and lower and upper quartiles. Asterisks indicate significance levels: + <0.1, * <0.05, ** <0.01, *** <0.001.

The ANOVA with the factors PREDICTED OUTCOME and STIMULATION on cue-locked neural responses revealed a significant interaction of both factors in a cluster located at the right dorsolateral prefrontal cortex (dlPFC) regions between 170 and 190 ms (*p*-cluster = 0.043; see Figure 5). Neural activity to cues that preceded the prediction of a loss was enhanced after excitatory compared to after inhibitory stimulation [*post-hoc t*-test: *t*_(32)_ = 3.11, *p* = 0.002, *d* = 0.57]. No significant difference was observed in response to cues that preceded gain predictions, *t*_(32)_ = −0.50, *p* = 0.688. No main effect of PREDICTED OUTCOME and STIMULATION reached significance.

##### 3.1.2.2. REWARD PROBABILITY-by-STIMULATION ANOVA

**Figure 6:**
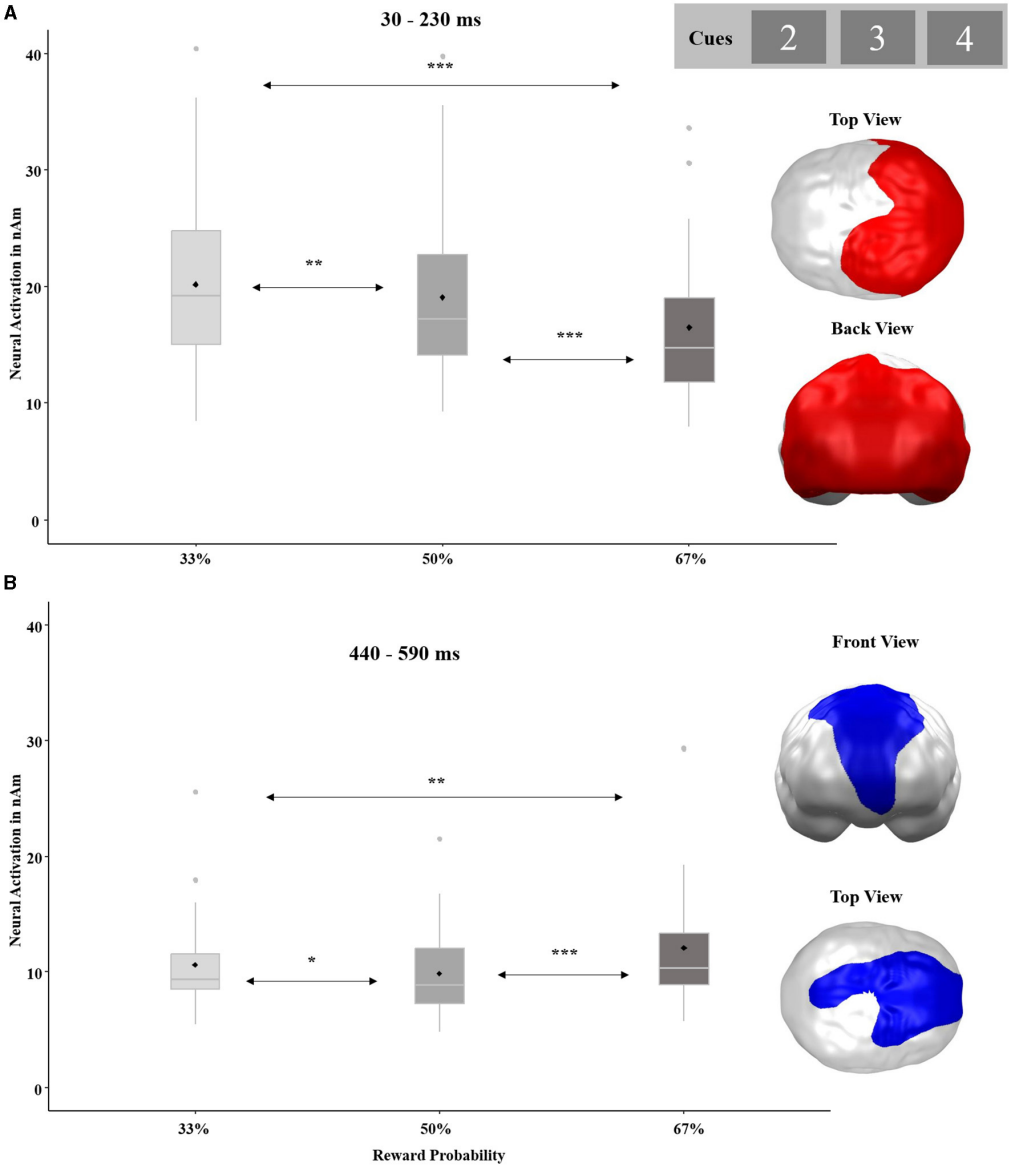
Significant spatio-temporal clusters featuring a main effect for REWARD PROBABILITY (33, 50, and 67%) on cue-locked neural responses. **(A)** Posterior brain regions with decreasing neural activations in response to increasing reward probability. **(B)** Frontoparietal brain regions with strongest neural activations in response to the highest reward probability. Topographies of effects observed in L2-MNE were projected on standard 3D brain models for visualization. Boxplots indicate means (black dot), medians (gray line), and lower and upper quartiles. Asterisks indicate significance levels: + <0.1, * <0.05, ** <0.01, *** <0.001.

The ANOVA with the factors REWARD PROBABILITY and STIMULATION revealed two significant main effects of REWARD PROBABILITY with opposite patterns (see Figure 6). The first spatio-temporal cluster covered the entire posterior brain and became significant in an early time interval between 30 and 230 ms (*p*-cluster < 0.001). The strongest activation in this cluster occurred in response to the lowest 33% chance of gain and decreased with increasing reward probability. *Post-hoc* tests revealed significant differences among all conditions [33 vs. 50%: *t*_(32)_ = −3.92, *p* = 0.001, *d* = −0.68; 50 vs. 67%: *t*_(32)_ = −9.91, *p* < 0.001, *d* = −1.73; 33 vs. 67%: *t*_(32)_ = −10.26, *p* < 0.001, *d* = −1.79]. The second cluster with an opposite gradient stretched from the prefrontal to parietal regions and occurred rather late between 440 and 590 ms (*p*-cluster = 0.031). In this cluster, the greatest activation was observed in response to the 67% cue, while smaller activations emerged in response to the 33% cue and even smaller activations after the 50% cue. Again, all conditions differed significantly from each other [33 vs. 50%: *t*_(32)_ = −2.22, *p* = 0.017, *d* = −0.39; 50 vs. 67%: *t*_(32)_ = 4.25, *p* < 0.001, *d* = 0.79; 33 vs. 67%: *t*_(32)_ = 3.15, *p* = 0.002, *d* = 0.55]. No main effect of STIMULATION or interaction effect of STIMULATION by REWARD PROBABILITY reached significance in this analysis.

#### 3.1.3. ANS correlates (cue-locked)

Both the REWARD PROBABILITY-by-STIMULATION ANOVA and the PREDICTED OUTCOME-by-STIMULATION ANOVA revealed (trend-)significant temporal clusters for the main effect of STIMULATION ranging from 710 to 1,060 ms (*p*-cluster = 0.052) and 750 to 1,120 ms (*p*-cluster = 0.017), respectively.

**Figure 7:**
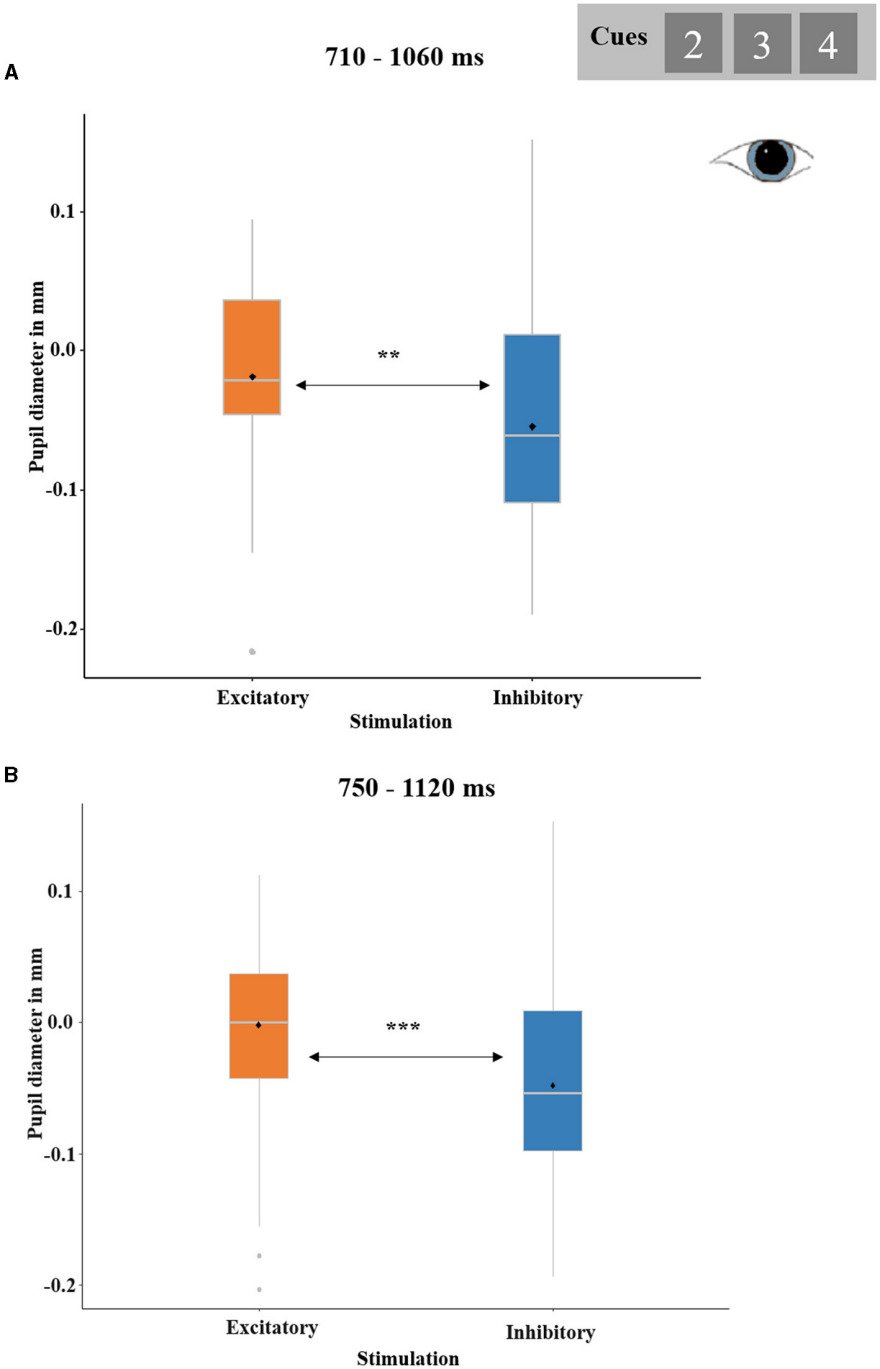
Significant effect of STIMULATION (excitatory and inhibitory) on cue-locked pupil responses. **(A)** Temporal cluster showing relative pupil dilations after excitatory compared to inhibitory stimulation in the analysis REWARD PROBABILITY (33, 50, and 67%) by STIMULATION. The depicted pupil diameters were baseline-adjusted (enabling negative values). Boxplots indicate means (black dot), medians (gray line), and lower and upper quartiles. **(B)** Temporal cluster showing relative pupil dilations after excitatory compared to inhibitory stimulation in the analysis PREDICTED OUTCOME (gain predicted, loss predicted) by STIMULATION.

In both clusters, pupil dilations were greater after excitatory as compared to after inhibitory stimulation [*t*_(28)_ = 2.65, *p* = 0.007, *d* = 0.49; *t*_(28)_ = 3.33, *p* < 0.001, *d* = 0.62, respectively; see Figure 7]. No other effects reached significance.

### 3.2. Reward processing and its modulation via vmPFC-tDCS

#### 3.2.1. Behavioral correlates

**Figure 8:**
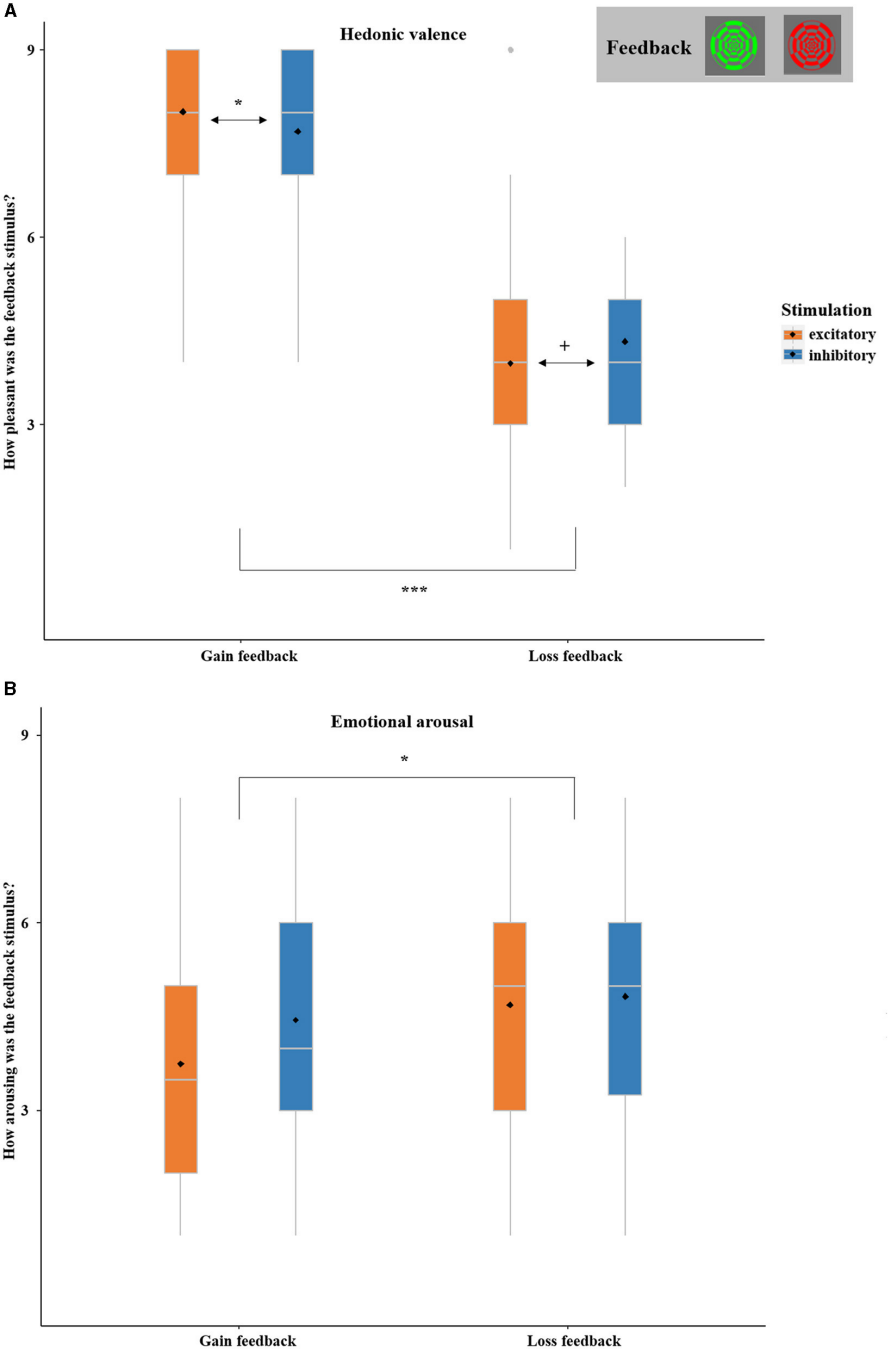
**(A)** SAM ratings of hedonic valence (1 = highly unpleasant, 9 = highly pleasant) of gain and loss stimuli as collected at the end of each session (not trial-wise). Gains were rated as more positive and losses were by trend rated as more negative after excitatory in comparison to inhibitory vmPFC stimulation. **(B)** SAM ratings of emotional arousal (1 = low arousal, 9 = high arousal) of gain and loss stimuli as collected at the end of each session (not trial-wise). Losses were overall rated as more arousing than gains. Boxplots indicate means (black dot), medians (gray line), and lower and upper quartiles. Asterisks indicate significance levels: + <0.1, * <0.05, ** <0.01, *** <0.001.

The ANOVA with the factors FEEDBACK and STIMULATION confirmed the anticipated main effect of FEEDBACK, with more positive ratings for gains in comparison to losses [*F*_(1,36)_ = 140.05, *p* < 0.001, η^2^ = 0.98]. While the main effect of STIMULATION was insignificant [*F*_(1,36)_ = 0.026, *p* = 0.812], the interaction effect of FEEDBACK by STIMULATION reached significance [*F*_(1,36)_ = 4.36, *p* = 0.043, η^2^ = 0.11; Figure 8A]. After excitatory stimulation, as compared to after inhibitory stimulation, gains were rated as more positive [*t*_(37)_ = 1.71, *p* = 0.048, *d* = 0.91] and losses were by trend rated as more negative [*t*_(37)_ = −1.67, *p* = 0.052, *d* = −0.88].

The ANOVA with the factors FEEDBACK and STIMULATION showed an unexpected main effect of FEEDBACK, with higher arousal ratings for losses compared to gains [*F*_(1,36)_ = 4.44, *p* = 0.042, η^2^ = 0.11]. The main effect of STIMULATION [*F*_(1,36)_ = 2.39, *p* = 0.131] and the interaction of FEEDBACK-by-STIMULATION did not reach significance [*F*_(1,36)_ = 3.528, *p* = 0.069; see Figure 8B]. For details see Supplementary material 1.1.

#### 3.2.2. Neural correlates (feedback-locked)

##### 3.2.2.1. RECEIVED OUTCOME *t*-test

**Figure 9:**
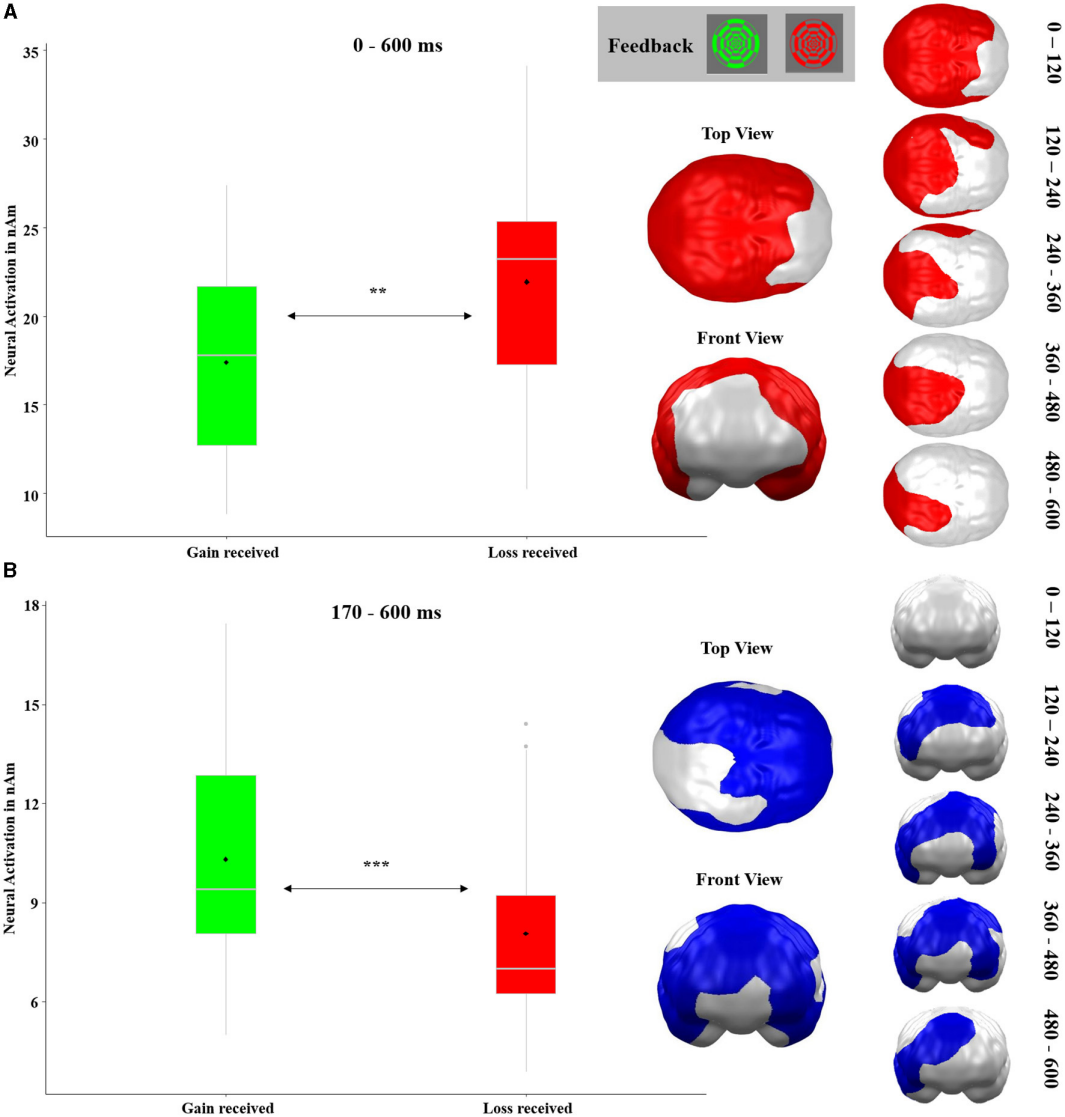
Significant spatio-temporal clusters featuring main effects of RECEIVED OUTCOME (gain received and loss received) on feedback-locked neural responses as revealed by *t*-test. **(A)** Posterior brain region with stronger neural responses to losses as compared to gains. **(B)** Frontal, temporal, parietal, and left occipital brain regions with greater activations in response to gains compared to losses. This cluster was originally constituted of two clusters. For the original clusters and their development across time, see Supplementary material 1.2. Graphics show the maximal temporal and spatial extent of each cluster. Because some brain regions show temporally changing shifts of activity (loss>gain or gain>loss), some areas appear in both clusters. Topographies of effects observed in L2-MNE were projected on standard 3D brain models for visualization. Boxplots indicate means (black dot), medians (gray line), and lower and upper quartiles.

The *t*-test comparing neural responses toward gain vs. loss feedback revealed three significant spatio-temporal clusters. The first cluster with stronger reactions to losses as compared to gains stretched almost across the entire time interval (10–600 ms; It should be noted that, to prevent latency shifts, MEG data has been filtered in a forward-backward fashion. As backward filtering smears effects to time points preceding the “real” effects, smearing of very strong statistical effects might provoke significance even preceding the “real” onset of an effect.). The cluster covered all posterior temporal, parietal, and occipital brain regions (*p*-cluster < 0.001, see Figure 9A). The other two clusters which were localized more frontally and showed stronger reactions to gains compared to losses were merged since they appeared in strongly overlapping time intervals and partially overlapping regions (see Figure 9B; original clusters are displayed in Supplementary material 1.2). This merged cluster emerged at 170–600 ms in frontal, temporal, parietal, as well as left occipital areas (both clusters: *p*-cluster < 0.001).

##### 3.2.2.2. RECEIVED OUTCOME-by-PREDICTED OUTCOME-by-STIMULATION ANOVA

**Figure 10:**
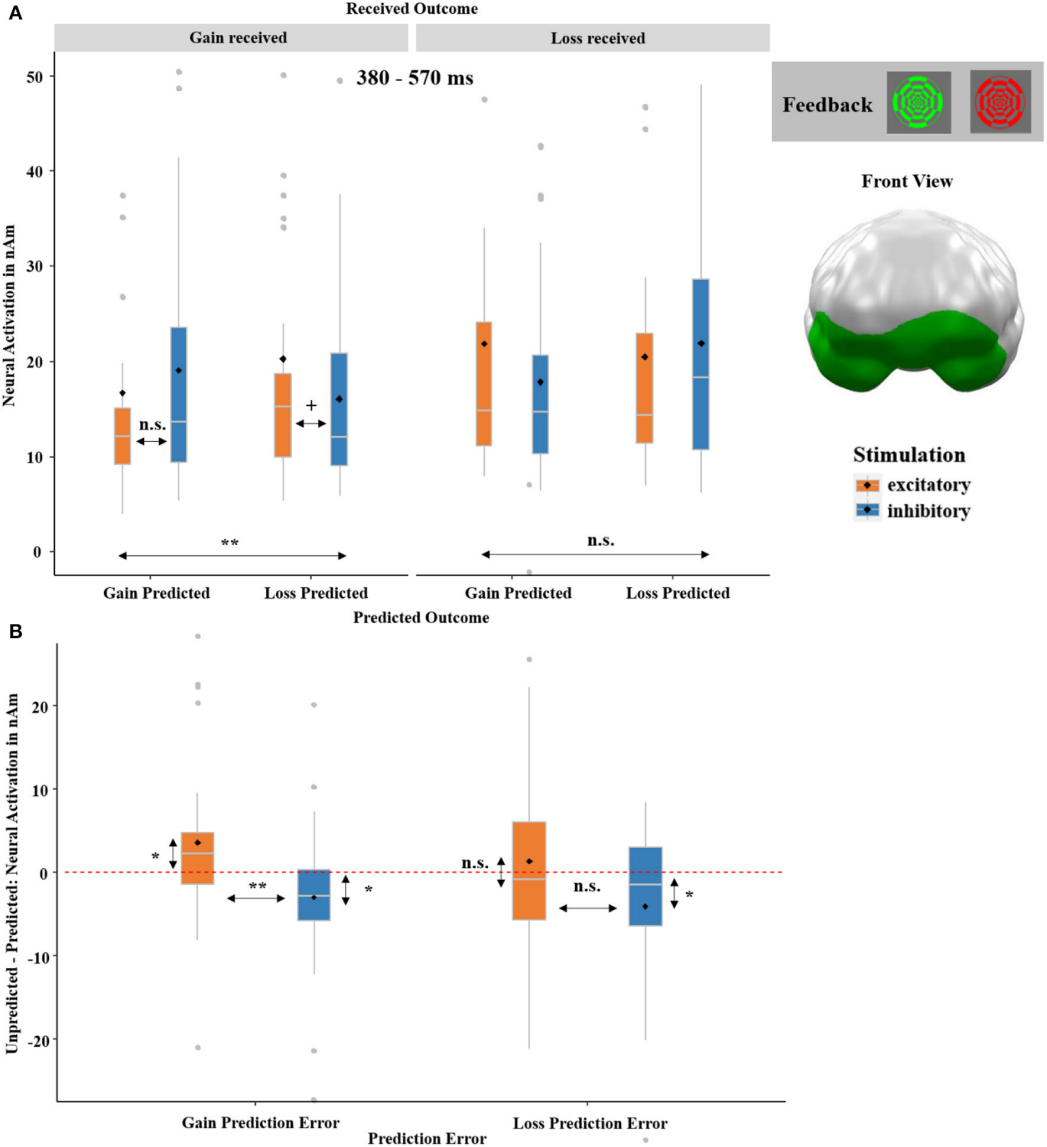
**(A)** Significant spatio-temporal cluster featuring a significant three-way interaction of RECEIVED OUTCOME (gain received and loss received)-by-PREDICTED OUTCOME (gain predicted, loss predicted)-by-STIMULATION (excitatory, inhibitory) on feedback-locked neural responses. **(B)** The three-way interaction was further separated into a gain and a loss prediction error (RPE). The gain +RPE was calculated, by subtracting the neural activation of trials in which a gain was predicted and a gain was received from the neural activation of trials in which a loss was predicted, but a gain was received (i.e., unpredicted *minus* predicted gain outcome). The loss -RPE was likewise calculated, by subtracting the neural activation of trials with predicted losses from the neural activation of trials with unpredicted losses (i.e., unpredicted *minus* predicted loss outcome). A significant difference with relatively increased neural activation for unpredicted compared to predicted outcomes was especially observed for gain +RPEs (i.e., unpredicted *minus* predicted gain outcome) after excitatory compared to after inhibitory tDCS, indicating relatively enhanced learning adjustments after excitatory stimulation. Individual RPEs can be compared to *zero*, here visualized via a red dashed horizontal line, representing identical processing of unpredicted and predicted outcomes. Topographies of effects observed in L2-MNE were projected on standard 3D brain models for visualization. Boxplots indicate means (black dot), medians (gray line), and lower and upper quartiles. Asterisks indicate significance levels: + <0.1, * <0.05, ** <0.01, *** <0.001.

The ANOVA calculated across feedback-locked neural responses revealed a significant three-way interaction of RECEIVED OUTCOME-by-PREDICTED OUTCOME-by-STIMULATION. This interaction emerged at 380–570 ms in ventromedial prefrontal areas (*p*-cluster = 0.044; see Figure 10A). We resolved this three-way interaction by calculating separate ANOVAs for received gain and received loss, respectively. This revealed an insignificant interaction of PREDICTED OUTCOME-by-STIMULATION after received losses [*F*_(1,32)_ = 2.98, *p* = 0.095, η^2^ = 0.09] and a corresponding significant interaction after received gains [*F*_(1,32)_ = 10.63, *p* = 0.003, η^2^ = 0.23]. The latter was primarily driven by the trend-significant difference between excitatory and inhibitory stimulation after received gains when a loss was predicted [*t*_(32)_ = 1.52, *p* = 0.069, *d* = 0.26].

Importantly, the three-way interaction indicated that the vmPFC stimulation modulated the processing of the received outcome (gain received and loss received) dependent on whether the individual prediction (gain predicted and loss predicted) matched the outcome or not. Thus, we resolved the three-way interaction further by looking at the effect of STIMULATION on gain and loss prediction errors (RPE). The gain +RPE was calculated by subtracting the neural activation of trials in which a gain was predicted and then received from the neural activation of trials in which a loss was predicted, but a gain was received (i.e., unpredicted minus predicted gain outcome). The loss -RPE was likewise calculated by subtracting the neural activation of trials with predicted losses from the neural activation of trials with unpredicted losses (i.e., unpredicted minus predicted loss outcome). Paired *t*-tests indicated a significant difference after excitatory as compared to inhibitory tDCS for gain +RPEs (see Figure 10B). As indicated by a comparison of individual RPEs against the test value *0*, the stimulation effect on gain +RPEs was driven by an increase of gain +RPEs after excitatory [*t*_(32)_ = 2.24, *p* = 0.032, *d* = 0.38] and a decrease of gain +RPEs after inhibitory stimulation [*t*_(32)_ = −2.17, *p* = 0.048, *d* = −0.30]. The stimulation effects on loss -RPEs were insignificant after inhibitory stimulation [*t*_(32)_ = −1.73, *p* = 0.093].

##### 3.2.2.3. Exploratory follow-up analysis on choice of the door across trials

To examine whether vmPFC-tDCS did not only modulate neural prediction errors but also affected future choice behavior, we calculated an additional analysis with which we investigated the effect of the previous outcome, previous prediction, and vmPFC stimulation on the choice of door. Therefore, we recoded the choice of door on each individual trial depending on the choice of door on the preceding trial into the binary variable change of door (yes/no), which expressed that the choice of door changed or did not change in this trial compared to the preceding one. Subsequently, we calculated another logistic regression using the predictors PRECEDING OUTCOME (gain preceding and loss preceding), PRECEDING PREDICTION (correct prediction preceding and incorrect prediction preceding), and STIMULATION (excitatory and inhibitory) to analyze whether a change of door was performed or not.

This logistic regression on the change of door revealed a significant model [$\chi {{\;}^{\text{2}}}_{\left(3\right)}$ = 35.59, *p* < 0.001] and an insignificant main effect of PRECEDING OUTCOME (*z* = −1.71, *p* = 0.089). Interestingly, an interaction effect of PRECEDING OUTCOME-by-STIMULATION occurred as well (*z* = −2.18, *p* = 0.029, *OR* = 0.81). This effect was characterized by more door changes after losses in the excitatory compared to the inhibitory condition [$\chi {{\;}^{\text{2}}}_{\left(1\right)}$ = 7.74, *p* = 0.006, *OR* = 0.79], while the respective difference was insignificant after gains [$\chi {{\;}^{\text{2}}}_{\left(1\right)}$ = 0.80, *p* = 0.372]. Additionally, the interaction effect of PRECEDING PREDICTION-by-STIMULATION was significant (*z* = −1.96, *p* = 0.050, *OR* = 0.84). *Post-hoc* test revealed more door changes after unpredicted outcomes in the excitatory compared to the inhibitory condition [$\chi {{\;}^{\text{2}}}_{\left(1\right)}$ = 6.71, *p* = 0.009, *OR* = 0.88] and no differences after predicted outcomes [$\chi {{\;}^{\text{2}}}_{\left(1\right)}$ = 0.84, *p* = 0.357].

There was also a significant interaction of PREDICTED OUTCOME-by-STIMULATION in the ventromedial and left prefrontal cortex between 130 and 190 ms (*p*-cluster = 0.044). Details of this interaction are reported in the Supplementary material 1.3 since we did not have specific hypotheses concerning this effect.

### 3.3. ANS correlates (feedback-locked)

The ANOVA with the factors RECEIVED OUTCOME, PREDICTED OUTCOME, and STIMULATION revealed significant temporal clusters for the main effects of RECEIVED OUTCOME and STIMULATION.

The main effect for RECEIVED OUTCOME (see Figure 12A) with relatively dilated (or less constricted) pupils in response to gains in comparison to losses occurred between 380 ms and 1,280 ms (*p*-cluster < 0.001). Excitatory compared to inhibitory stimulation also resulted in relatively dilated pupils in a time interval between 310 ms and 580 ms (*p*-cluster = 0.021; see Figure 12B). No interaction effects reached significance.

### 3.4. Mood effects

#### 3.4.1. PANAS

Neither the main effect of STIMULATION [*F*_(1,36)_ = 0.30, *p* = 0.586] nor the interaction effect of AFFECT by STIMULATION [*F*_(1,36)_ = 0.60, *p* = 0.443] was significant. Separate analyses conducted for positive and negative affect also revealed no effects of stimulation on mood [Pos: *F*_(1,36)_ = 0.47, *p* = 0.498; Neg: *F*_(1,36)_ = 0.46, *p* = 0.500].

## 4. Discussion

Our goal was to investigate the causal role of vmPFC activity in reward prediction (cue) as well as reward processing (feedback) by employing an established gambling paradigm and modulating vmPFC activity non-invasively via tDCS. In our within-subjects design, we applied excitatory and inhibitory tDC stimulation of the vmPFC on two different days in all participants. We found evidence that the vmPFC is involved in both reward prediction and reward processing, which can be consistently observed on the behavioral, the autonomous nervous system, and the neural levels. Regarding reward prediction (cue-locked analyses), vmPFC excitation as compared to inhibition resulted in tentatively more optimistic reward predictions, enhanced dlPFC activation to cues preceding loss predictions, and overall enhanced pupil dilations to cues. Furthermore, we observed greater medial prefrontal activity in response to a greater chance to win and greater posterior brain activation in response to a greater chance to lose. Regarding reward processing (feedback-locked analyses), vmPFC excitation as compared to inhibition resulted in increased pleasantness of gains, enhanced vmPFC activation to especially unpredicted gains (i.e., gain +RPEs), less perseveration in choice behavior after loss feedback and incorrect predictions, as well as overall enhanced pupil dilations to feedback. In addition, we observed greater frontal brain activation to gains, suggesting that RewP is more dominant than FN and greater posterior brain activation to losses, indicating arousal.

### 4.1. Reward prediction

As anticipated, a general overestimation of reward occurred, which even increased with higher reward probability. This optimism bias is in line with previous findings and is commonly found in humans (Sharot, 2011). In the analysis of self-reported reward prediction with both reward probability and stimulation as predictors, the effect of stimulation was masked. However, when we chose a hypothesis-driven approach with stimulation as the only predictor, we could show increased self-reported reward prediction after vmPFC excitation (see Figure 4). Although this can only be regarded as tentative evidence for the effect of vmPFC stimulation on behavior, this finding should stimulate further investigations. For example, it would be interesting to know whether vmPFC-tDCS could increase reward prediction in clinical populations suffering from aberrant reward expectancy (e.g., in mood disorders), while inhibitory stimulation might serve as a model for a “depression-like” more negatively biased pattern.

On the neural level, we were able to support our behavioral results. We found an interaction of PREDICTED OUTCOME and STIMULATION in the right dlPFC, which indicates enhanced neural activity before loss predictions after excitatory compared to after inhibitory stimulation (see Figure 5). As the dlPFC plays a cardinal role in the inhibition of fear responses and emotion regulation (Rehbein et al., 2015; Wessing et al., 2017; Notzon et al., 2018; Roesmann et al., 2021), this effect can well be interpreted as preventive downregulation of neural responding when a loss is expected. The result that excitatory tDCS of the vmPFC increases the inhibition of loss processing has also been shown previously by our group. Investigating the perception of the framing effect after vmPFC-tDCS, we found that vmPFC excitation improved the inhibition of risky decisions and irrational negative feedback ratings (Kroker et al., 2022).

Second, we found a significant main effect of REWARD PROBABILITY (i.e., neural response to the cues) in posterior brain areas and at rather early latencies (30–230 ms). In this cluster (see Figure 6A), the neural activity increased with a greater probability of loss, irrespective of stimulation. Along the gambling paradigm, participants learned that the cues predict the probability of loss or gain feedback, respectively (i.e., associative learning). Following this early and mid-latency posterior effect with increasing neural processing of increasingly loss-predicting cues, cues predicting gains generated relatively enhanced neural processing in predominantly prefrontal brain regions at a rather late time interval (440–590 ms; see Figure 6B). These increased gain responses in PFC regions of later time intervals dovetail with previous findings concerning the contribution of medial prefrontal areas to enhanced reward prediction or inhibition of excessive loss processing, respectively (Hiser and Koenigs, 2018; Cao et al., 2019; Fryer et al., 2021). As predictive reward cues have been shown to elicit similar neural responses as the reward stimuli themselves (Holroyd et al., 2011), the enhanced prefrontal activity to a greater chance to win and the enhanced posterior activity to a greater chance to lose (see Figures 6A, B) can be understood as predictive indicators preceding the enhanced prefrontal activation to actual gains and the enhanced posterior activation to actual losses (see Figures 9A, B).

The pupil data showed a significant main effect of stimulation at 710 to 1,063 ms and 750 to 1,120 ms, respectively, with relatively larger pupil diameters after excitatory than after inhibitory stimulation (see Figure 7). This finding fits the findings of the reward processing phase, as in this case outcome and stimulation induced similar effects: Excitatory stimulation and monetary gains both induced greater pupil diameters indicating that vmPFC excitation evokes similar pupil effects as rewards. For a more detailed discussion see below.

### 4.2. Processing of received reward

**Figure 11:**
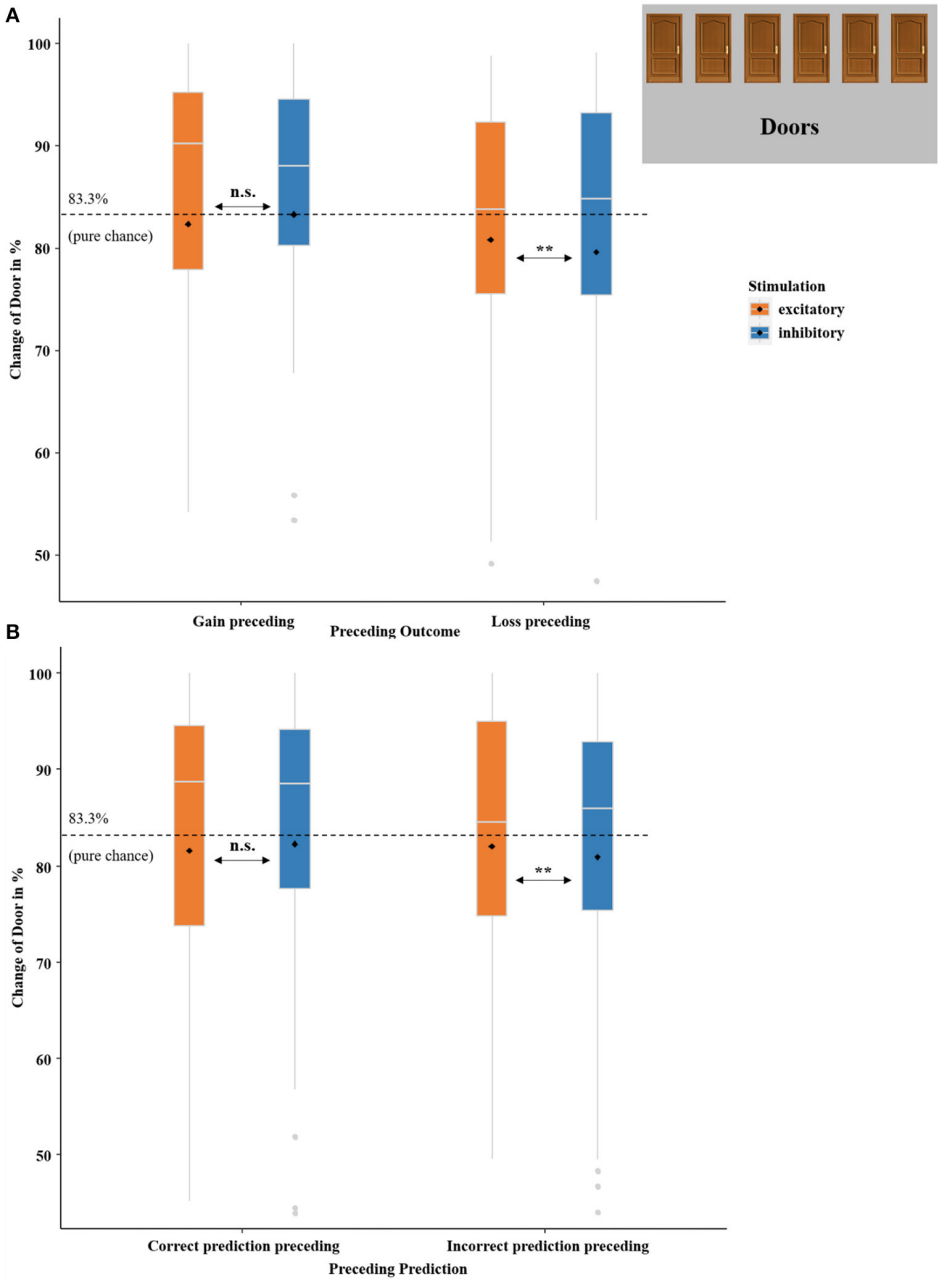
Change of door in percent. **(A)** Boxplots indicate the probability of a change in the choice of door compared to the preceding trial in dependency of PRECEDING OUTCOME (gain preceding and loss preceding) and STIMULATION (excitatory and inhibitory). **(B)** Boxplots indicate the probability of a change in the choice of door compared to the preceding trial in dependency of PRECEDING PREDICTION (correct prediction preceding and incorrect prediction preceding) and STIMULATION (excitatory and inhibitory). The dashed black lines indicate pure chance (83.3%) that a change of door selection occurs. Boxplots indicate means (black dot), medians (gray line), and lower and upper quartiles. Asterisks indicate significance levels: + <0.1, * <0.05, ** <0.01, *** <0.001.

Convergent to the ratings of gain and loss expectations (cues), valence ratings of received gain and loss feedback were also modulated by stimulation, although the feedback ratings (in contrast to the expectation rating) were assessed only once at the end of each session: here gains were rated as more positively and losses were rated by trend as more negatively after excitatory in comparison to inhibitory vmPFC stimulation (see Figure 8A). This effect might probably relate strongly to the proposed vmPFC function in estimating reward valuation based on prior experience (Cao et al., 2019). In a real-world environment, more positive ratings of gains after excitatory stimulation would lead to a more frequent choice of this option in future, while a more negative rating of losses after vmPFC excitation would lead to a less frequent choice of this option, overall indicating more efficient affective learning after excitatory or relatively diminished affective learning after inhibitory stimulation. Interestingly, we observe exactly this pattern in the reward prediction phase. After losses in the excitatory condition, the door is more likely to be changed and after gains, the door is more likely to be retained (whereby the latter is not significant) compared to inhibitory stimulation. As this behavioral pattern can be regarded as a more adaptive way to respond to received feedback, it indicates a more sophisticated processing of the consequences that follow the behavior after vmPFC excitation (see Figure 11). This is also supported by the interaction effect of stimulation by previous prediction, as participants respond more flexibly to incorrect predictions with a door change. Although there is no correct or incorrect choice of door (since wins and losses were randomly distributed across the individual doors), a behavior more closely resembling this chance distribution can be viewed as more adaptive. Additionally, the analysis of door changes revealed a trend for preceding outcomes characterized by fewer behavioral changes after losses compared to gains. This behavior is rather irrational since after losses, one should seek alternative options that offer better outcomes. However, this behavior is well-known and might be explained with the so-called *gambler's fallacy*: After (multiple) losses, gamblers expect to win in a “fair” game, even though each outcome is independent of the previous one (Tversky and Kahneman, 1971; Xue et al., 2011). Therefore, participants are more likely to stay with the same door (“But now, finally, there must be a gain behind this door”). Importantly, however, excitatory vmPFC-tDCS reduces this bias relative to inhibitory stimulation as can be seen in the interaction of stimulation with preceding outcome.

These behavioral findings are also well in line with the neural three-way RECEIVED OUTCOME-by-PREDICTED OUTCOME-by-STIMULATION interaction (see Figure 10), where we measured greater activity after unpredicted compared to predicted (especially gain) outcomes following excitatory stimulation. Both findings point to the enhanced valuation of positive outcomes after vmPFC excitation and suggest greater behavioral flexibility and facilitated learning from preceding outcomes after excitatory tDCS.

The SAM ratings of the feedback stimuli (see Figure 8B) revealed overall increased emotional arousal for loss compared to gain feedback, although the gain-to-loss ratio of 2:1 was explicitly chosen to balance the salience of gains and losses (Kahneman and Tversky, 1979; Tversky and Kahneman, 1992). This residual effect might be due to the fact that our sample consisted mainly of relatively low-income students who might have perceived monetary losses as relatively even more arousing. However, it might also reflect the instance that the checkerboard feedback stimuli were only indirectly associated with the monetary 2:1 ratio of loss vs. gain based on the basic introduction and the following SAM rating (see Figure 1) and might have thus evoked a residual inherent negativity bias.^3^

In the neural analysis of outcomes (gains vs. losses), we obtained an extensive cluster with a main effect of RECEIVED OUTCOME (see Figure 9A) covering the whole brain, except PFC regions, and effectively over the entire time interval (10−600 ms). This strong and sustained effect can again well be explained by gain/loss arousal differences since greater neural activations were seen for losses which were rated as relatively more arousing (Lang et al., 1998). Furthermore, the location and great spatio-temporal extent of the cluster might be explained by the perceptual differences between the stimuli. This is because the difference between red and green could have contributed to both the occipital localization and the large arousal, as the colors red and green have a strong signaling character. More interesting for our hypotheses is the second cluster (see Figure 9B), which might be regarded as a magnetic equivalent of the RewP. In all PFC and parietal cortex regions—with the exception of the stimulated vmPFC region—stronger neural activations occurred for gains in comparison to losses starting at 170 ms. This cluster resembles in topographical and temporal distribution, one of the electric RewP (Carlson et al., 2011; Proudfit, 2015), although the cluster had a much greater temporal extent from 170 to 600 ms than the typical RewP component. Nevertheless, the distribution of this cluster suggests that in the present study, the RewP is more dominant than the FN, as here the reward-related effects dominate the typical FN/RewP time range in the respective regions (see Figure 9B). It should be noted that this pattern is present in the cue phase as well, since here reward-related effects occur in prefrontal areas (see Figure 6B) and loss-related effects emerge in posterior areas (see Figure 6A). This interpretation must be viewed cautiously, however, because we are considering the results in source space, whereas the FN/RewP are typically studied in sensor space.

The three-way interaction of RECEIVED OUTCOME-by-PREDICTED OUTCOME-by-STIMULATION, again occurring in the vmPFC and within a rather later time interval (380–570 ms), relates to the assumed function of the vmPFC. Here, greater activations occurred in response to unpredicted outcomes (after both actual gains and losses) following excitatory compared to inhibitory stimulation. This pattern indicates that the vmPFC is involved in a re-evaluation of reward probability, which is also suggested by the behavioral data (SAM-rating; see Figure 8A). The rather late onset of this effect further supports this interpretation of a higher-order cognitive process. Finally, it is likewise well-encouraged by other neuroimaging (Hiser and Koenigs, 2018; Kroker et al., 2022) and lesion studies (Pujara et al., 2016). The process of re-evaluation supposedly appears very pronounced for predicted losses, which is already evident in reward predictions (i.e., cue processing; see Figure 5) and was also present in our previous gambling study. In this study, we showed that a reduced framing effect after excitatory compared to inhibitory vmPFC-tDCS was mainly driven by reduced loss processing (Kroker et al., 2022). Therefore, it stands to reason that the vmPFC is (co-) responsible for inhibiting loss expectations or loss responses resulting in greater reward predictions and improved learning after vmPFC excitation.

**Figure 12:**
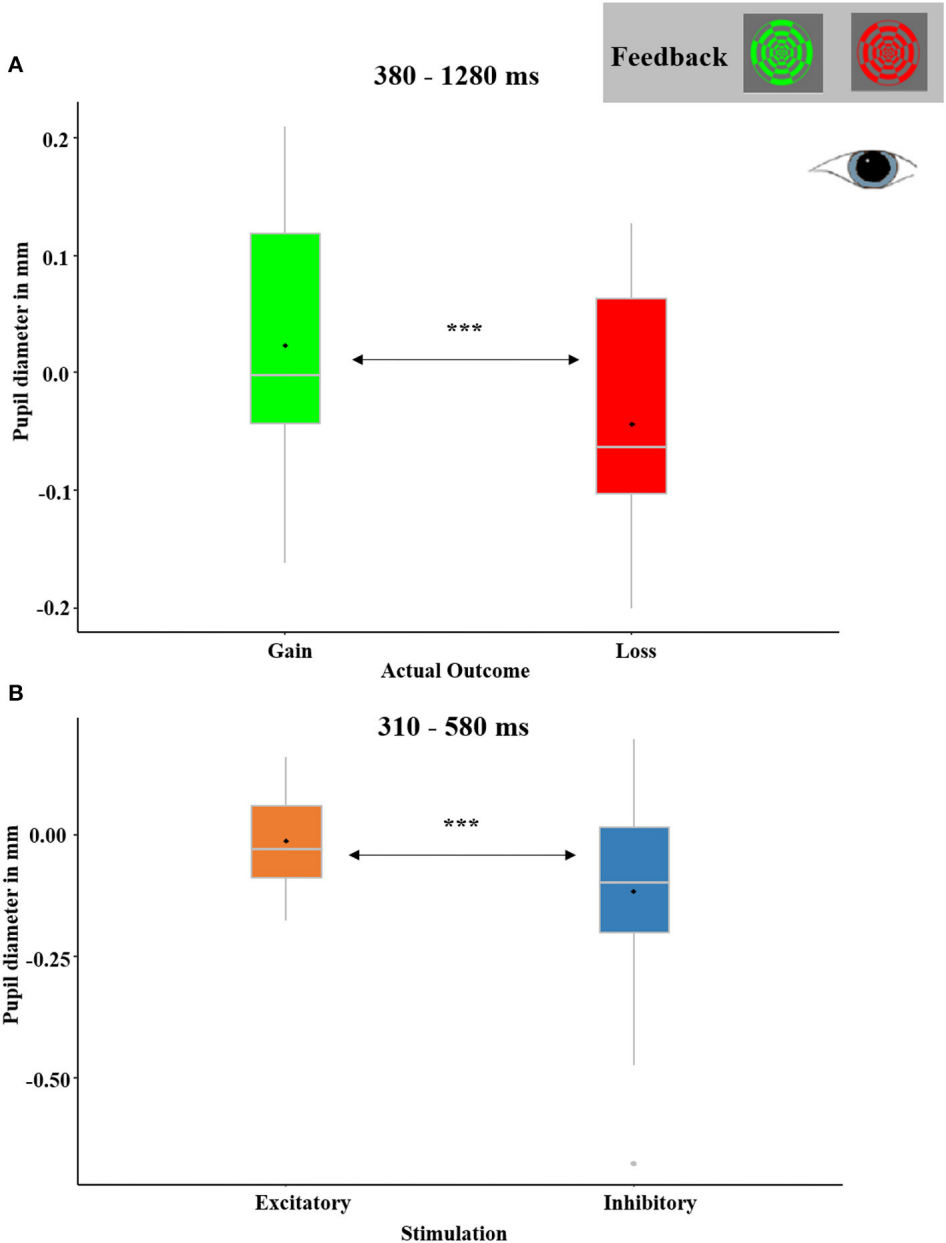
**(A)** Significant temporal cluster of feedback-locked pupil diameters with a main effect of RECEIVED OUTCOME (gain received and loss received). Pupils were relatively dilated in response to gain feedback than loss feedback between 380 and 1,280 ms. The depicted pupil diameters were baseline-adjusted (enabling negative values). **(B)** Significant temporal cluster of pupil diameters with a main effect of STIMULATION (excitatory and inhibitory). Pupils were relatively dilated after excitatory as compared to after inhibitory stimulation between 310 and 580 ms. Boxplots indicate means (black dot), medians (gray line), and lower and upper quartiles.

In addition to our findings on the effects of tDCS in the reward prediction phase, we likewise found interesting effects affecting pupil data in the reward processing phase. It is known from previous studies that pupil diameters increase in response to rewards (Bijleveld et al., 2009; Koelewijn et al., 2018). We have not only found significant temporal clusters with more dilated pupils for gains than losses (see Figure 12A) but also more dilated pupils after excitatory compared to inhibitory stimulation (see Figure 12B). It should be noted that continued enhanced pupil dilations when a reward is expected have been associated with the maintenance of a high arousal level that is beneficial for fully processing a reward once it is received (e.g., Rudebeck et al., 2014). As the vmPFC has been shown to regulate physiological arousal (e.g., Zhang et al., 2014), it could be speculated that vmPFC excitation enhanced physiological arousal as indicated by pupil dilation. The physiological arousal could have boosted the prediction and the processing of rewards.

In addition to all these new insights into vmPFC functioning and non-invasive brain stimulation, some aspects require further consideration. First, to guarantee the successful blinding of participants to the stimulation conditions and to reduce inter-individual variance, we here opted for a within-subjects-design and sacrificed the comparison with a sham condition (i.e., participants easily detect the difference between active and sham tDCS while both active conditions are typically indistinguishable). All conclusions regarding causal functionality and modulating capability of the vmPFC remain unaffected. Nevertheless, it remains to be resolved whether inhibitory stimulation solely might evoke temporary vmPFC dysfunctions in healthy controls leading to a reduced optimism bias, for instance, reported for depressive patients, and/or if excitatory stimulation could strengthen the optimism bias even in healthy participants. To investigate this question, follow-up studies should use between designs comparing the effects of excitatory vs. sham vmPFC-tDCS in healthy participants. Second, we observed an unexpected main effect of FEEDBACK affecting the SAM arousal ratings indicating greater arousal levels after losses. This could also be due to the fact that we recorded the valence and arousal ratings only once after the MEG measurement and not after each trial, which might have distorted the measurement. However, increased arousal seems to be mirrored in the neural data as well, since we found extensive clusters in posterior brain regions with stronger activations for losses or increasing probability of loss. These clusters partly masked prefrontal reward effects (gain > loss) in an ANOVA. However, we solved this problem by calculating a *t*-test that did not influence the interpretation of prefrontal reward effects. Furthermore, interaction effects involving stimulation occurred not only in the vmPFC but also in neighboring more superior prefrontal regions (dlPFC). This indicates that the vmPFC is part of a network responsible for reward prediction/processing and that vmPFC stimulation likewise influences reward processing in these areas. Another aspect to consider is that we surveyed reward prediction after the feedback presentation, although other time points would also have been conceivable. However, we chose this time point as we wanted to avoid interfering effects of the query on stimulus processing in the MEG. Finally, despite increased pupil diameters after excitatory compared to inhibitory stimulation for both cue and feedback processing, we did not find modulatory effects of vmPFC stimulation on pupil reactivity driven by reward probability, outcome, or prediction. This might be due to the fact that the stimulus timing has been optimized for the MEG as our principal physiological measure and might have been too fast for the relatively slower pupil reactions.

#### 4.2.1. Reward prediction error (RPE) signaling

A closer look at the gain and loss prediction errors (+RPE/–RPE) suggests a potential involvement in more optimistic reward predictions after excitatory compared to inhibitory stimulation. Stronger reactions to unpredicted gains (+RPEs) compared to unpredicted losses (–RPEs) after vmPFC excitation as compared to vmPFC inhibition suggest a relatively stronger updating of reward probability after gains than losses. This mechanism, in turn, may have led to the more optimistic reward predictions in the excitatory condition. Stronger gain prediction errors might also partly explain why reward-based learning strategies are often more efficient than punishment-based learning strategies (Wächter et al., 2009; Cunningham and Cramer, 2013). Most importantly, the enhanced activation to unpredicted gains (+RPE) after vmPFC excitation as compared to vmPFC inhibition observed here might present an important mechanism behind the positivity bias previously observed in tDCS studies conducted at our lab (Junghofer et al., 2017; Winker et al., 2018, 2019, 2020; Kroker et al., 2022) and rTMS findings from other groups (Ryan et al., 2022). Indeed, excitatory vmPFC-tDCS might strengthen the valuation of especially unpredicted positive events, which leads to a more positive appraisal of events and facilitates behavioral responses directed at rewards.

## 5. Conclusion

Our results provide evidence for the causal role of the vmPFC in reward prediction and reward processing by using non-invasive brain stimulation. We found significant effects of vmPFC stimulation in the central (MEG-data) and autonomous nervous system (pupil data) as well as in the behavioral data, all pointing in the same direction. The results suggest a dual function of the vmPFC consisting of (1) a positivity bias (partly mediated by inhibition of loss processing) that can be seen in reward prediction and early reward processing and (2) a more sophisticated component that appears to re-evaluate reward probability at a later processing step. An overarching model explaining these findings in later time intervals is delivered by Hiser and Koenigs (2018), suggesting that the anticipation of rewards in the vmPFC is predominately driven by efficient reinforcement learning from prior experience (i.e., elaborate reward processing to maximize rewards in future). In sum, these findings significantly expand our understanding of how the vmPFC functions in the prediction and processing of rewards and show that the further use of tDCS in reward research is highly promising.

## Data availability statement

The raw data supporting the conclusions of this article will be made available by the authors, without undue reservation.

## Ethics statement

The studies involving human participants were reviewed and approved by Ethics Committee of the Medical Faculty of the University of Münster. The patients/participants provided their written informed consent to participate in this study.

## Author contributions

All authors listed have made a substantial, direct, and intellectual contribution to the work and approved it for publication.

## Footnotes

^1^The actual gain outcomes were rounded to multiples of 5 (35, 50, and 65%) and thus slightly deviated from the reward probabilities indicated by the number of doors (33, 50, and 67%). This deviation was introduced to increase the number of trials in each individual cell and to allow for even trial numbers (e.g., 28, 40, and 52 gain trials out of 80, respectively), though the reward probabilities indicated by the number of doors might have been more accurately represented by another trial number (e.g., 27, 40, and 53 gain trials out of 80, respectively).

^2^In an analysis with all three factors, some conditions would have not more than 14 trials or even less, which is not adequate for a reliable signal-to-noise ratio. For example, for Cue 2, there were 28 gain trials and if participants would have predicted gains and losses equally often, this would result in 14 trials in this condition.

^3^As event related magnetic fields (in contrast to hemodynamic reactions) show very distinct sensory correlates to different visual stimuli (e.g., the digits 30 vs. 15), we here opted for this indirect association and avoided a direct presentation of the monetary feedback values.
