# Supplementary material for: Non-invasive stimulation reveals ventromedial prefrontal cortex function in reward prediction and reward processing
Source: Front Neurosci. 2023 Aug 15;17:1219029. doi: 10.3389/fnins.2023.1219029 (PMC10465130; doi:10.3389/fnins.2023.1219029)
Supplement: Supplementary file 1 [file Data_Sheet_1.docx]

**Supplementary Materials (SM):**

## **SM1. Further results concerning reward processing**

## **SM1.1 Details regarding the subjective ratings of the gain and loss stimuli**

**Table SM1**

SAM-ratings in dependency of experimental conditions

| **SAM-Valence-ratings** | | | | | | | | | | | | | | | | | | |  |  |
| --- | --- | --- | --- | --- | --- | --- | --- | --- | --- | --- | --- | --- | --- | --- | --- | --- | --- | --- | --- | --- |
|  | **Excitatory** | |  | | | **Inhibitory** | | |  | |  | |  | |  | |  |  |  |  |
|  | **M** | | **SD** | | | **M** | | | **SD** | | ***F*** | | **df_n** | | **df_d** | | ***p*** | ***η2*** |  |  |
| **Condition** |  |  | | |  |  | |  | | |  | | |  | |  | |  | | |
| Gain | 8.00 | | 1.18 | | | 7.71 | | | 1.47 | | - | | - | | - | | - | - |  |  |
| Loss | 3.95 | | 1.70 | | | 4.29 | | | 1.77 | | - | | - | | - | | - | - |  |  |
| Received Outcome | - | | - | | | **-** | | | **-** | | 140.06 | | 1 | | 36 | | **< .001** | 0.796 |  |  |
| vmPFC-Stimulation | 5.98 | | 1.44 | | | 6.00 | | | 1.62 | | 0.03 | | 1 | | 36 | | 0.812 | 0.001 |  |  |
| Stimulation x Received Outcome | - | | - | | | - | | | - | | 4.36 | | 1 | | 36 | | **0.044** | 0.108 |  |  |
| **SAM-Arousal-ratings** | | | | | | | | | | | | | | | | | | | |  |
|  | **Excitatory** | | |  | | | **Inhibitory** | | |  | |  |  | |  | |  |  | |  |
|  | **M** | | | **SD** | | | **M** | | | **SD** | | ***F*** | **df_n** | | **df_d** | | ***p*** | ***η2*** | |  |
| **Condition** |  |  | | |  |  | |  | | |  | | |  | |  | |  | | |
| Gain | 3.70 | | | 2.11 | | | 4.43 | | | 2.10 | | - | - | | - | | - | - | |  |
| Loss | 4.74 | | | 2.01 | | | 4.83 | | | 1.66 | | - | - | | - | | - | - | |  |
| Received Outcome | - | | | - | | | **-** | | | **-** | | 4.44 | 1 | | 36 | | **0.042** | 0.101 | |  |
| vmPFC-Stimulation | 4.22 | | | 2.05 | | | 4.63 | | | 1.88 | | 2.39 | 1 | | 36 | | 0.131 | 0.062 | |  |
| Stimulation x Received Outcome | - | | | - | | | - | | | - | | 3.53 | 1 | | 36 | | 0.069 | 0.089 | |  |

*Note.* The *p*-values indicate significance of the coefficients in a repeated-measures 2x2 ANOVA.

**Table SM1.** SAM-ratings of hedonic valence (1 = highly unpleasant, 9 = highly pleasant) and of emotional arousal (1 = low arousal, 9 = high arousal) of gain and loss stimuli as collected at the end of each session (not trial-wise). Gains were rated as more positive and losses were rated as more negative after excitatory in comparison to inhibitory vmPFC stimulation. Losses were overall rated as more arousing than gains and arousal of gains was relatively reduced after excitatory stimulation.

**Table SM2**

Number of subjects for each measurement

|  | **Behavior** | **MEG** | | **Pupil** | | |  |  |  |  |  |  |
| --- | --- | --- | --- | --- | --- | --- | --- | --- | --- | --- | --- | --- |
| **Reward Prediction** |  |  |  | |  |  | |  | |  |  |  |
| *N* | 41 | 33 | | 29 | | |  | |  | | |  |
| **Reward Processing** |  |  | |  | | |  | |  | | |  |
| *N* | 38 | 33 | | 29 | | |  | |  | | |  |

*Note.* All behavioral and neural results were qualitatively equivalent if the smallest shared sample of 29 participants was used.

## **SM1.2 Cue-locked and feedback-locked Global Power plots displaying the time course of MEG and pupil data**

## **
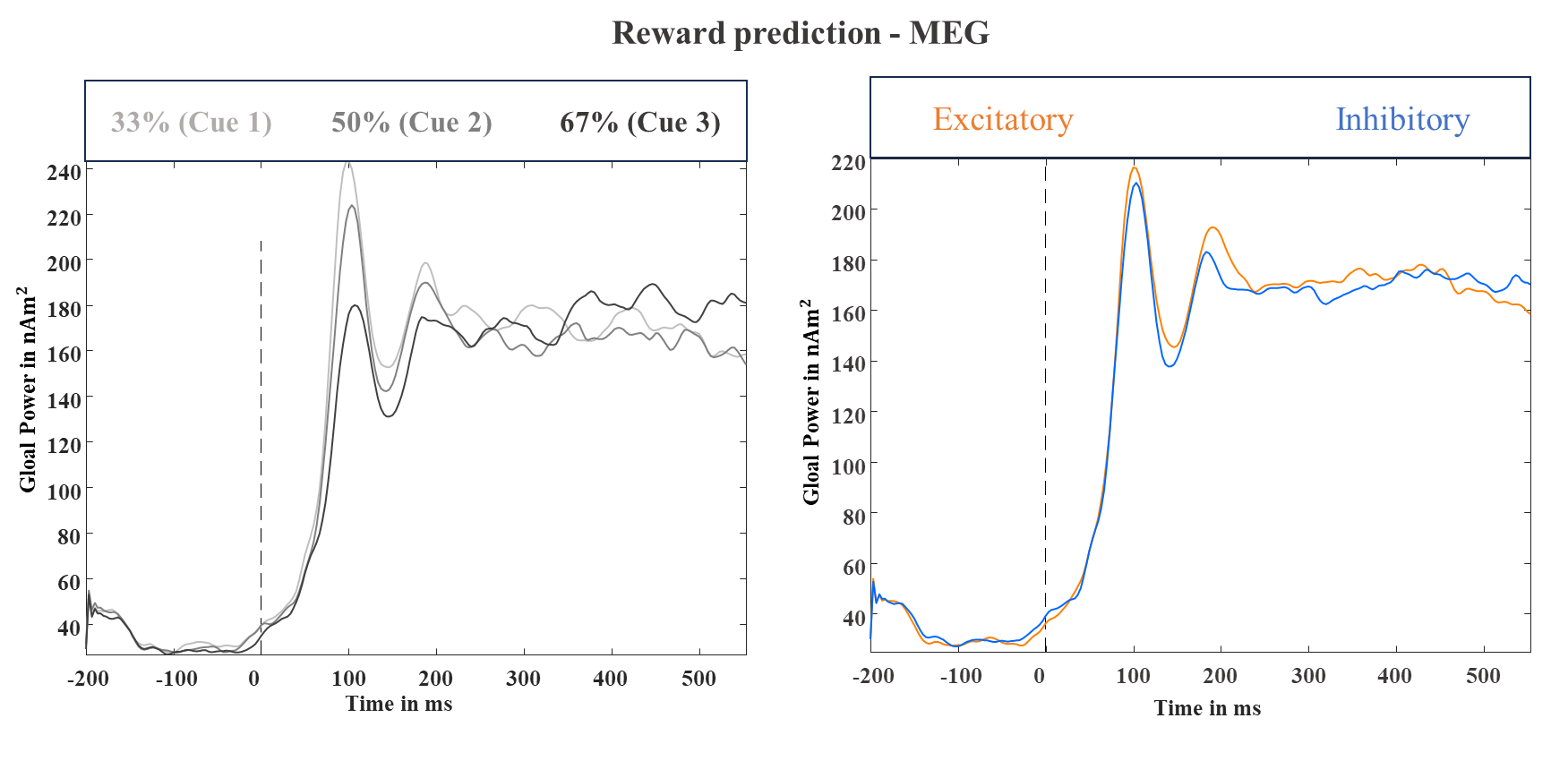
**

## **
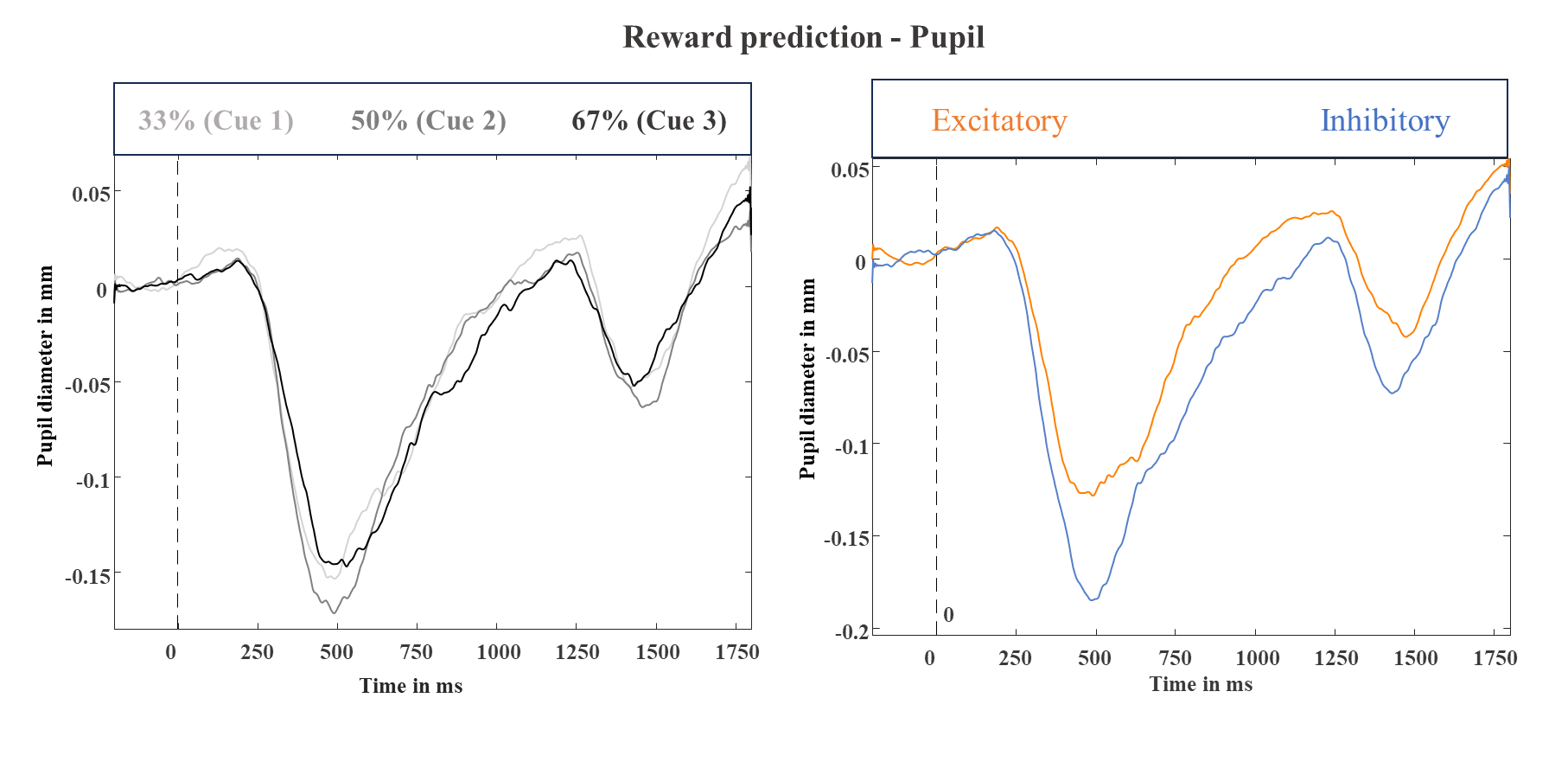
**


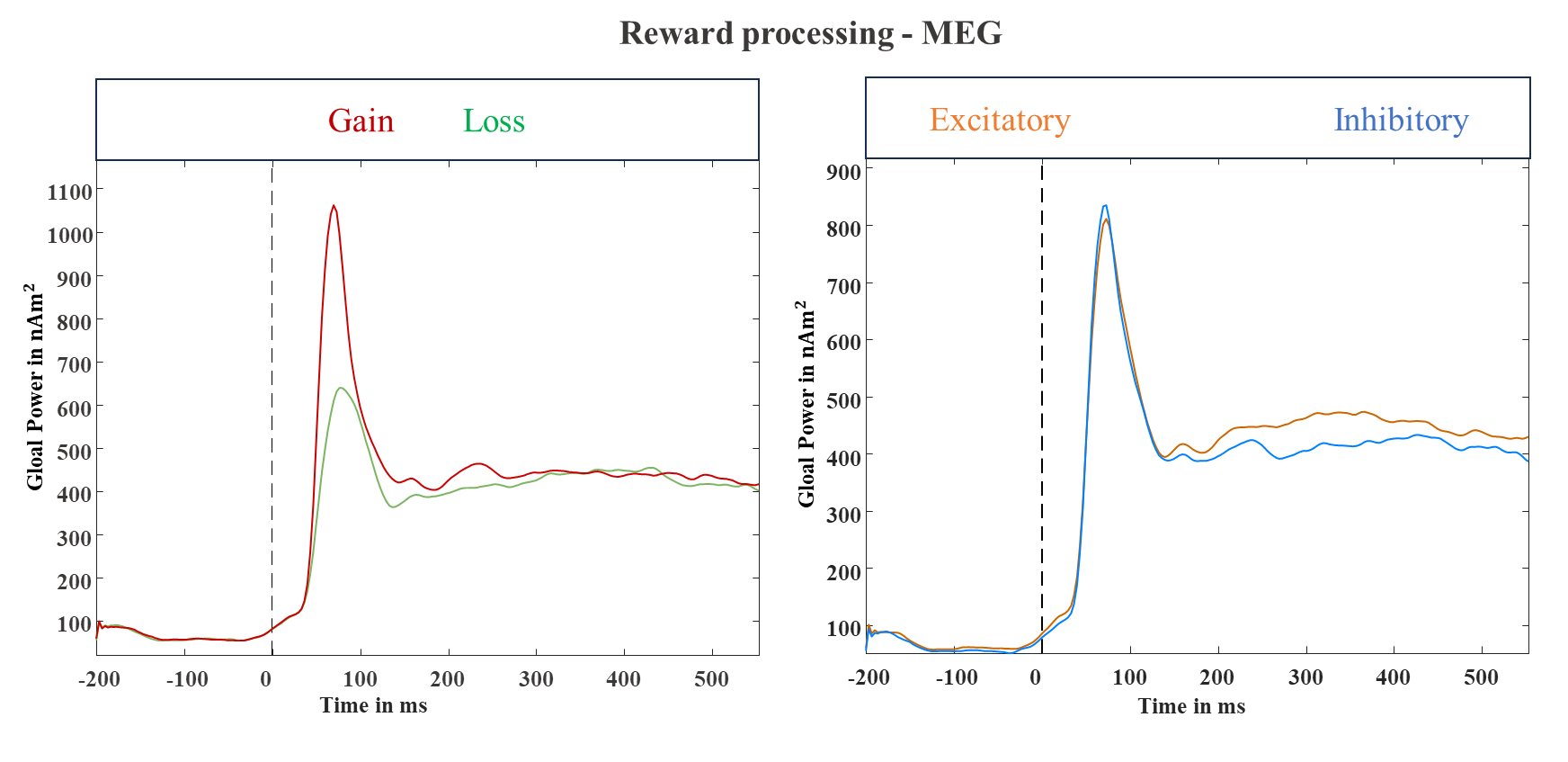


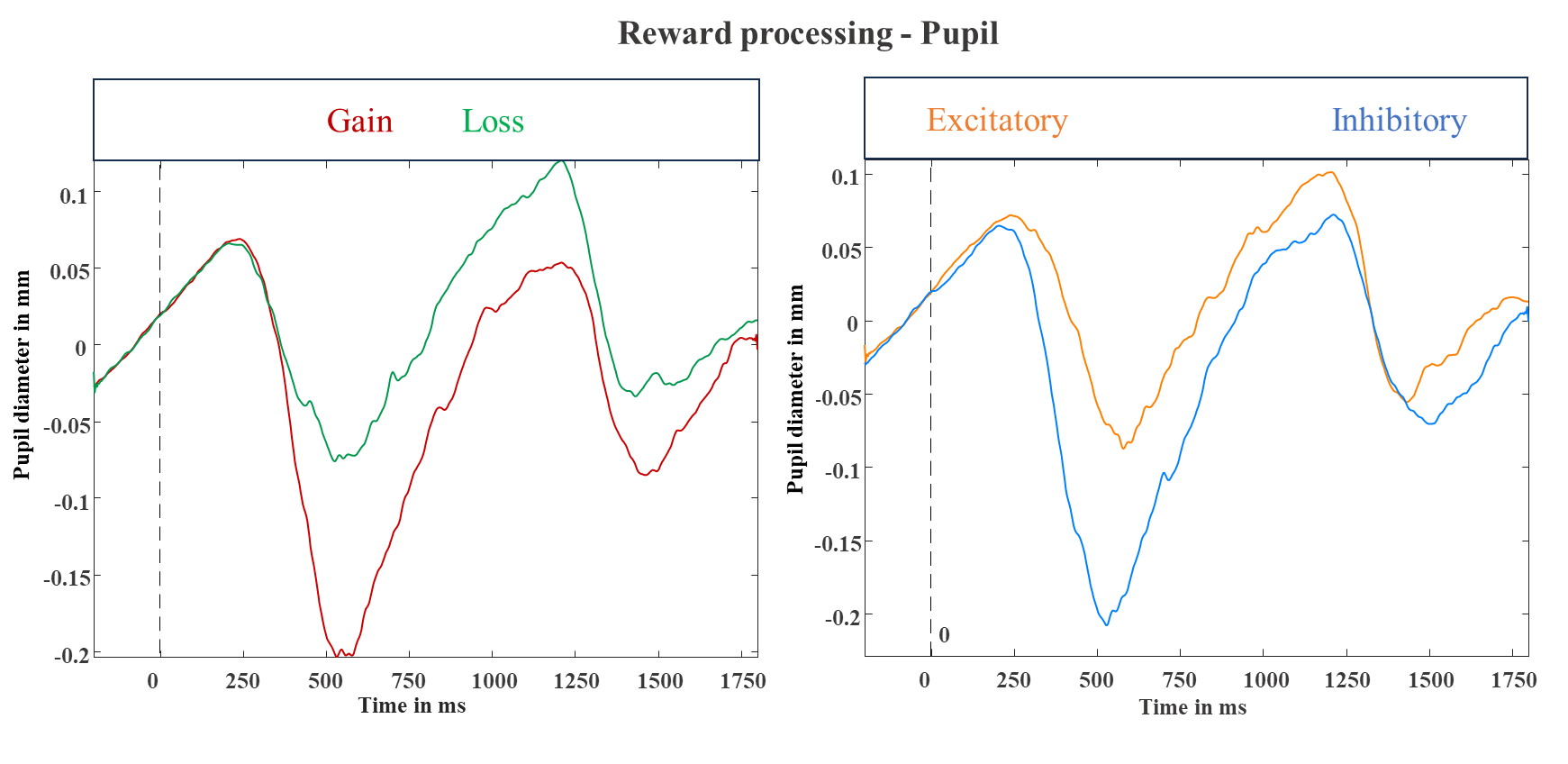


## *Figure SM1.* Global Power plots for the cue-locked (reward prediction; upper panels) and feedback-locked (reward processing; lower panels) MEG and pupil data for the different cue, feedback, and stimulation conditions.

## **SM1.3 Main Effect of RECEIVED OUTCOME (feedback-locked neural responses)**

In order to depict the main effect for RECEIVED OUTCOME as clearly as possible, this figure exhibits the spatial extent of the three clusters over the time course. In contrast to cluster A (see main text), the post-hoc tests of cluster B (*t*(32) = 10.99, *p* < 0.001) and cluster C (*t*(32) = 5.96, *p* < 0.001) revealed stronger neural activations after gains compared to after losses.


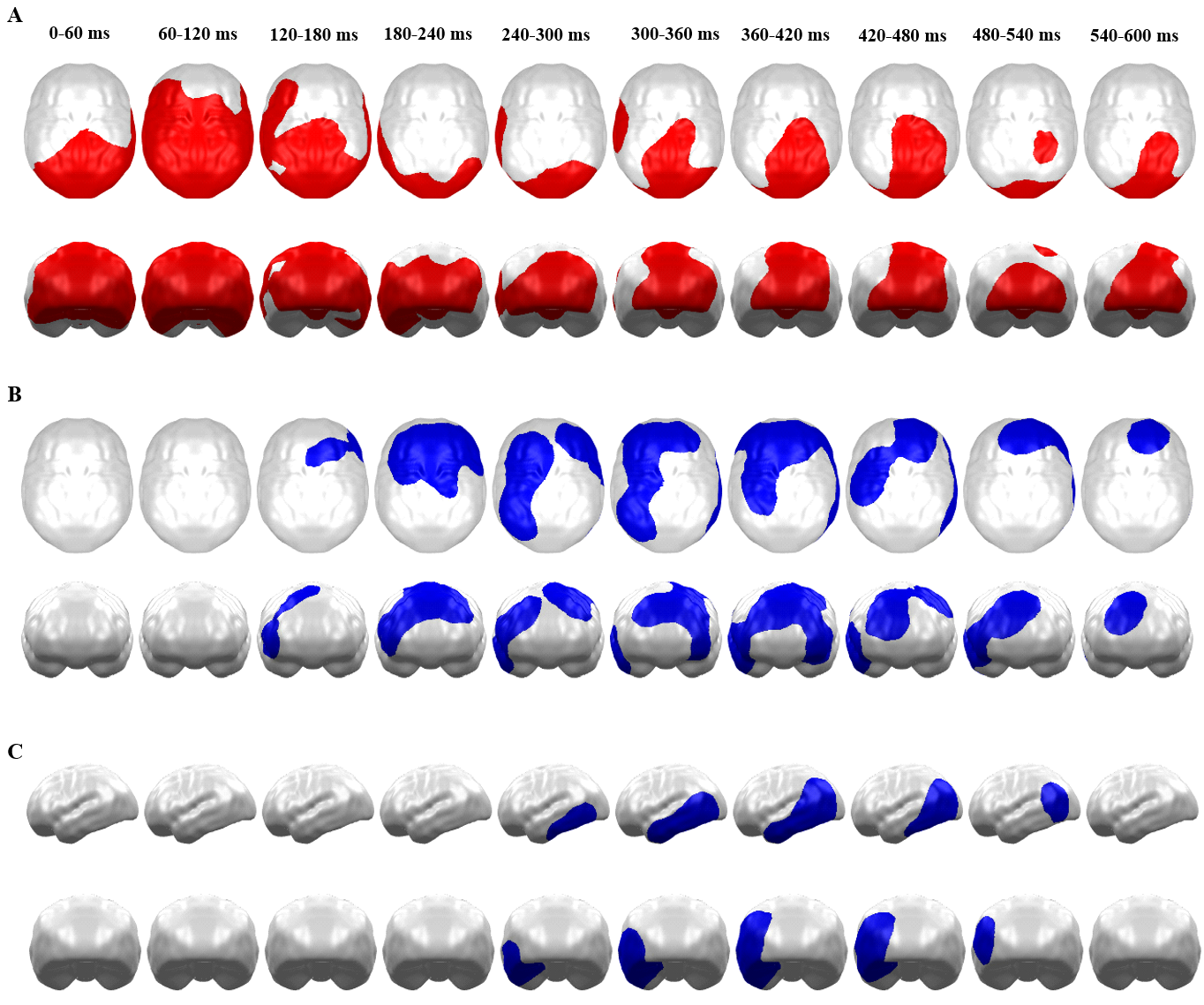
*Figure SM2.* Time courses of the three significant spatio-temporal clusters with a main effect of RECEIVED OUTCOME as revealed by a t-test. These clusters are shown here to clarify that some brain regions show changing shifts of difference activities (leading to spatial overlap of clusters showing opposite effects e.g. gain>loss at early latencies followed by loss>gain at later time intervals). Red clusters indicate stronger neural activations in response to stimuli that are associated with negative compared to positive outcomes, while blue clusters indicate stronger neural activations in response to stimuli that are associated with positive compared to negative outcomes. Cluster B and C were merged together and visualized in figure 9B of the main text.

## **SM1.4 Interaction effect of PREDICTED OUTCOME and STIMULATION (feedback-locked neural responses)**

There was a significant interaction of PREDICTED OUTCOME by STIMULATION in the ventromedial and left prefrontal cortex between 130 to 190 ms (*p*-cluster = 0.044). In this cluster, stronger activations occurred in response to predicted losses after excitatory compared to after inhibitory stimulation (*t*(32) = 3.55, *p* < 0.001), while the pattern was reversed, though not significant, for predicted gains (*t*(32) = -1.50, *p* = 0.139).


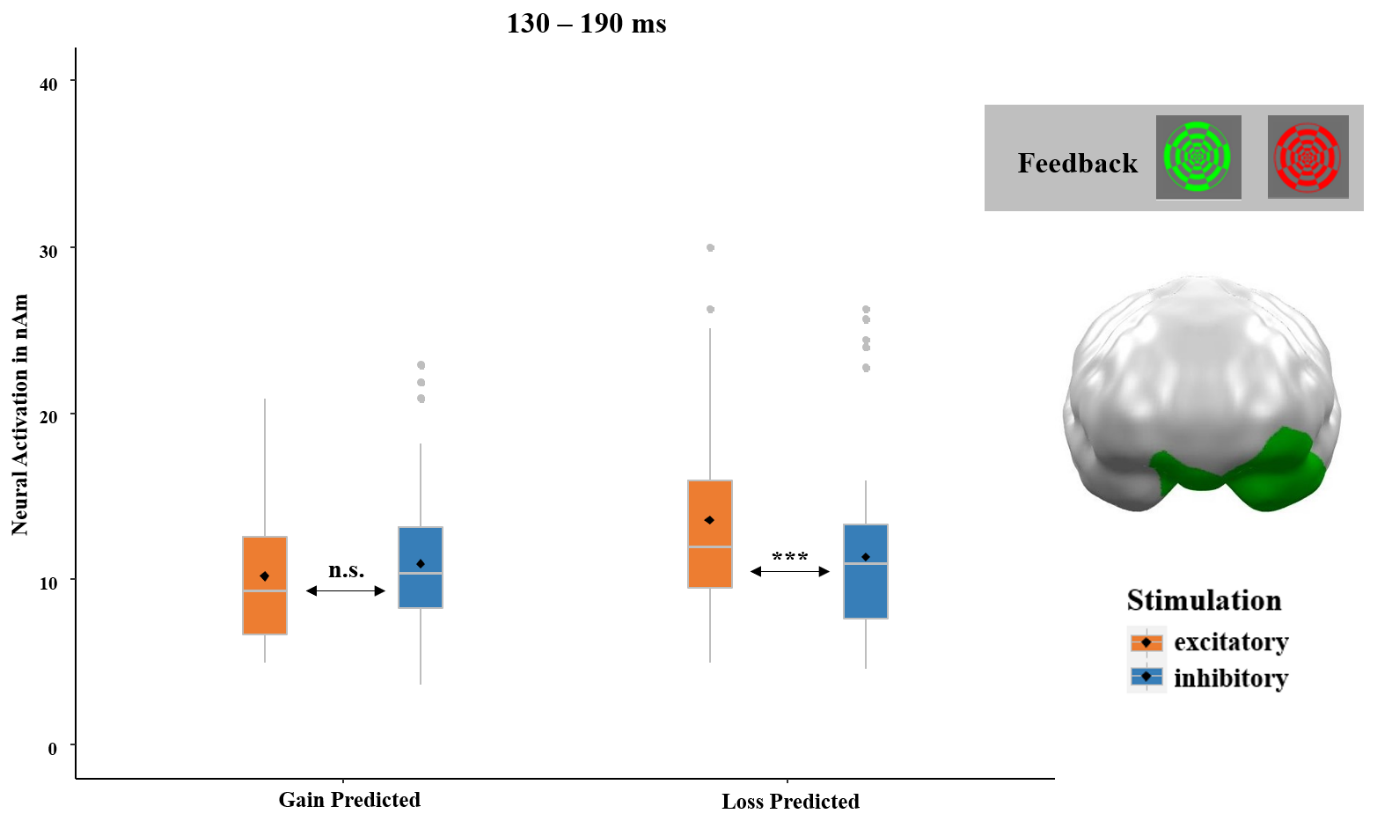
 *Figure SM3.* Significant spatio-temporal cluster in anterior brain areas featuring a significant interaction of PREDICTED OUTCOME (gain predicted, loss predicted) by STIMULATION (excitatory, inhibitory) covering left ventro-medial prefrontal cortices. Predicted losses evoked stronger activations after excitatory than after inhibitory stimulation.

Boxplots indicate means (black dot), medians (grey line) and lower and upper quartiles. Asterisks indicate significance levels: + < 0.1, * < 0.05, ** < 0.01, *** < 0.001.

## **SM2. Discussion**

The interaction effect of PREDICTED OUTCOME by STIMULATION in the vmPFC between 130 and 190 ms (see figure SM2) is in line with the idea that vmPFC-excitation can also enhance the inhibition of loss aversion as seen within dlPFC regions in an overlapping time interval (170 - 190 ms) during cue processing as well (see figure 5, main text). As discussed for the cues, one might conclude that this cluster reflects enhanced downregulation of (predicted) losses after vmPFC excitation, which can be seen as a vmPFC-function and occurs in early time ranges.
